# High‐Order Nonlinear Photonic Crystal with Ordered Structures for Giant Enhancement of Frequency Tripling

**DOI:** 10.1002/advs.202524069

**Published:** 2026-02-11

**Authors:** Xiaotian Guo, Qiaoling Han, Fei Liang, Bin Zhang, Dazhi Lu, Haohai Yu, Huaijin Zhang

**Affiliations:** ^1^ State Key Laboratory of Crystal Materials and Institute of Crystal Materials Shandong University Jinan China; ^2^ School of Physics Shandong University Jinan China

**Keywords:** frequency tripling, Nonlinear optical, phase matchingphotonic crystal, YAG

## Abstract

Nonlinear optical conversion can generate new laser frequency which is difficult to achieve by direct lasing. Till now, second‐order nonlinearity with χ^(2)^, e.g. frequency doubling, has been well developed with birefringent phase‐matching for the frequency dispersion compensation. Beyond χ^(2)^, third‐order χ^(3)^ nonlinearity can triple the optical frequency directly and allow multi‐photon entanglement, which is helpful to reduce the device size and improve the capacity of on‐chip photonics. However, triple‐photon suffers from the extremely low efficiency (10^−10^∼10^−8^) due to the difficulty of compensating optical dispersion at large frequency difference, as well as small χ^(3)^ coefficients. Here, we propose a high‐order nonlinear photonic crystal with artificially ordered structures for regulating phase compensation and boosting the third‐order conversion efficiency. Taking Y_3_Al_5_O_12_ crystal as an example, we realize the efficient frequency tripling in meta‐YAG with a conversion efficiency of 4.5×10^−3^, which is six orders of magnitude stronger than that of conventional YAG crystal. Moreover, a wide spectral tunability from 331 to 356 nm is achieved in the meta‐YAG, indicating its great potential for broadband triple‐photon generation. This study provides valuable insights into regulating all χ^(3)^‐active nonlinear optical materials and opens the way to new frontiers on multi‐photon entanglement in the nonlinear photonic crystal platform.

## Graphical abstract

A meta‐YAG crystal with artificially order/disorder structures was fabricated to realize the efficient third‐harmonic generation at 343 nm with a conversion efficiency of 4.5×10^−3^, surpassing six orders of magnitude compared to bulk YAG crystal. Moreover, a wide spectral tunability from 331 to 356 nm was also achieved in the meta‐YAG, indicating its great potential for broadband laser conversion.

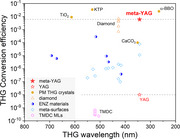

## 1 Introduction

Nonlinear optics is the study of phenomena that emerge as a result of the modified optical properties under high‐intensity light [1, 2, 3, 4, 5, 6, 7, 8]. Since the first discovery of frequency doubling in quartz [9], nonlinear optical effects have been a hot spot in the optical community and play a significant role in the field of quantum photonics and opto‐electronics [10, 11, 12, 13, 14]. On the basis of quadratic nonlinearity χ^(2)^, the extended laser spectra at various wavelengths were developed by second‐harmonic generation (SHG), optical parametric oscillation (OPO), *etc*, which has been widely utilized in many fields, comprising laser display, optical communication, laser manufacturing, and so forth [15, 16, 17, 18, 19]. Especially in the new era of quantum optics [1, 20], the entangled photon source could be created by spontaneous parametric down conversion (SPDC), emitting two correlated low‐energy photons in an optical crystal pumped by one high‐energy photon, which are themselves entangled. At present, many χ^(2)^‐based nonlinear crystals [21, 22, 23, 24] including β‐BBO, PPKTP, and PPLN, have been successfully applied in quantum optics experiments to generate the two‐photon polarized entangled state and squeezed state.

Differing from quadratic nonlinearity with the prerequisite of broken inversion symmetry, cubic nonlinearity with χ^(3)^ exists in all optical materials. On the basis of χ^(3)^ nonlinearity, the spontaneous four‐wave mixing (SFWM) process has been developed in silicon [25], silicon nitride [26], and gallium nitride [27]. In 1962, the first third‐harmonic generation (THG) was observed in CaCO_3_ crystal by Terhune [28]. Frequency‐tripling in χ^(3)^ optical materials is very interesting in quantum optics [29, 30], because it is at the origin of intrinsic three‐body quantum phenomena such as Greenberger‐Horne‐Zeilinger photon entanglement [31]. Therefore, triple‐photon would bring some revolutionary change for new applications, e.g. quantum cryptography. However, efficient frequency tripling remains a real challenge because cubic coefficients χ^(3)^ are very small, which is several orders of magnitude lower than χ^(2)^. A few attempts have been made to enhance THG efficiency in χ^(3)^‐materials, such as index‐matched bulk crystals [32, 33, 34], phonon resonance [13], topological bands [35], epsilon‐near‐zero films [36, 37, 38], and dielectric meta‐surfaces [39, 40]. Nevertheless, limited by the giant phase mismatch, the overall THG efficiency is very low, usually at 10^−10^–10^−5^ [41]. Even in a few index‐matched crystals with high efficiency (η ∼ 10^−3^) [42, 43], these materials suffer from poor wavelength tuneability, which hinders their extensive applications in optoelectronics. Therefore, finding new χ^(3)^ nonlinear materials and technology becomes imperative for frequency tripling with high efficiency and wide tuneability concurrently.

Microstructure, that ordered structures with functional units, is a powerful paradigm for altering the optical properties of crystal materials. Designing the artificial structure at micrometers in a homogenous crystal could be helpful for improving the nonlinear conversion efficiency by phase compensation from the reciprocal lattice vectors. For example, periodic optical superlattices with χ^(2)^ nonlinearity have been realized in PPLN, PPKTP, (Ba, Ca)TiO_3_, and quartz crystal to ensure the efficient SHG [44, 45, 46, 47, 48]. However, this strategy has never been applied in cubic χ^(3)^ nonlinearity, such as direct THG. There are two possible reasons for the absent micro‐structured χ^(3)^‐materials. First, the third‐order χ^(3)^ coefficients are much smaller than χ^(2)^. If we select traditional nonlinear crystals without inversion symmetry, the contribution of cubic χ^(3)^ would be easily overshadowed by strong quadratic χ^(2)^ [34]. This case limits the alternative nonlinear optical crystals. Second, aiming at the same emitting wavelength, the refractive‐index dispersion in THG process is much larger than SHG [49]. This would bring a giant phase mismatch, corresponding to the requisite of increased reciprocal lattice vector and reduced period length. Therefore, designing micro‐structured materials with χ^(3)^ nonlinearity remains a great challenge.

Herein, we proposed a new microstructure strategy for boosting the third‐order conversion efficiency and enlarging spectral tuneability simultaneously. Taking the centrosymmetric Y_3_Al_5_O_12_ (YAG) crystal as a prototype, we fabricated the meta‐YAG by laser direct‐writing and verified the feasibility of periodic microstructures to achieve efficient THG. The periodic length was 5.28 µm in meta‐YAG and the emitting THG signal located at 343 nm in the UV region. The maximum output power was 5.8 mW with a high conversion efficiency of 4.5×10^−3^. To our knowledge, this is the highest THG efficiency in nonlinear photonic crystals, surpassing those surface nonlinearity in meta‐surfaces and films. Moreover, compared to index‐matched crystals with small angle acceptance, a tunable THG at 331‐356 nm was realized in the meta‐YAG, which could be assigned to the synergistic effect of phase fluctuation and angular tuning. This study provides a simple method to design and fabricate χ^(3)^‐based nonlinear optical materials, which paves the new way for highly‐efficient cubic optical frequency mixing in quantum optics and integrated on‐chip photonics.

## 2 Results and Discussion

### 2.1 Design Principle for χ^(3)^‐Based Nonlinear Photonic Crystal

**FIGURE 1:**
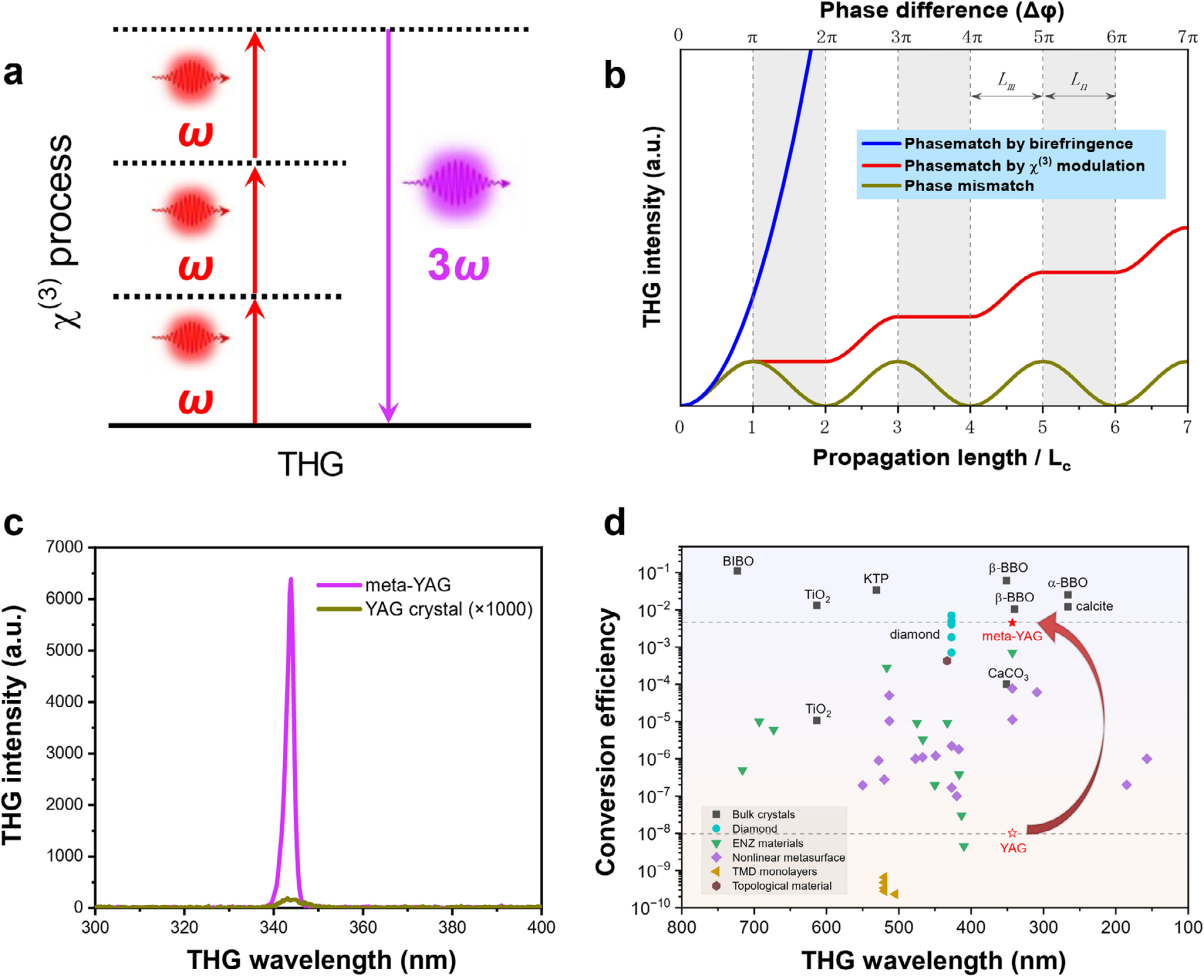
Direct THG process principle and schematic diagram of artificial microstructures. (a) Schematic of third‐order THG process, *ω*+*ω*+*ω*→3*ω*. (b) Principle of phase control of artificial microstructures. If the phase‐matching condition is satisfied, the TH intensity is shown as the blue curve. The green curve shows that energy cannot be effectively accumulated in the non‐phase‐match condition, and the red curve represents the frequency tripling conversion under the phase modulation by microstructure design. *L_m_* represents the length of the ordered region to produce an effective frequency conversion, and *L_n_* represents the length of the disordered regions in which periodic phase compensation is realized. In the disordered region, the χ^(3)^ nonlinearity was partially erased by laser direct‐writing. (c) The THG spectra of a meta‐YAG and a YAG crystal. Under 1030 nm laser excitation, the THG signal of the meta‐YAG is six orders of magnitude stronger than that of the YAG crystal. ×1000 means the THG intensity of YAG crystal is enlarged by 1000 times for clarity. (d) The comparison of THG efficiency of transition‐metal dichalcogenides (TMDCs) monolayer, dielectric meta‐surfaces, epsilon‐near‐zero (ENZ) films, non‐phase‐matched diamond, phase‐matched nonlinear crystals CaCO_3_, TiO_2_, KTP, α‐BBO, and our designed meta‐YAG crystal. A comprehensive comparison of THG in nonlinear materials was listed in Table S1.

Frequency tripling is a process in which the laser frequency is directly converted to three times the input light frequency by using nonlinear materials. The principle of the direct THG process is plotted in Figure 1a. In 1962, the first THG effect was experimentally realized in calcite [28] and then theoretically built by Bloembergen [50]. Based on the nonlinear optics theory, THG originated from the induced high‐order polarization by third‐order χ^(3)^ nonlinearity. Compared to the cascaded third‐harmonic wave from two χ^(2)^ processes, the efficiency of direct THG is usually weak owing to the small χ^(3)^ coefficients and strict phase‐matching condition.

Based on the basic equation of nonlinear frequency conversion, the complex amplitude of electric field *E*_3ω_(*z*) of THG light at the *z* position is described as Equation (1) by employing the slowly varying amplitude approximation [51],

(1) $$\frac{dE_{3\omega }\left(z\right)}{dz}=\frac{3i\omega \mu_{0}\varepsilon_{0}c}{2n_{3\omega }}\;\chi_{eff}^{\left(3\right)}E_{\omega }^{3}e^{i\Delta kz}$$

where *μ_0_* is the vacuum permeability, *ε_0_* is the vacuum permittivity, *ω* is the incident light frequency, *n_3ω_* is the refractive index of third‐harmonic wave in the nonlinear crystal, *i* and *c* are the complex unit and the speed of light in vacuum, respectively. $\chi_{eff}^{\left(3\right)}$ is effective third‐order coefficient of nonlinear crystal. *E*_ω_(*z*) is the complex amplitude of electric field of incident light at the *z* position. By adopting the small‐signal approximation, we consider that *E*_ω_ (*z*) =  *E*_ω_ (0) = *E*_ω_ . Owing to the refractive‐index dispersion in nonlinear crystals, there would be a phase difference defined as Δφ  =  Δ*k* · *z* between the fundamental‐wave (FW) and third‐harmonic (TH) wave, where Δ*k*  = *k*_3ω_  − 3*k*_ω_.

Substituting the relationship between light intensity and amplitude, the expression of the TH intensity *I_3ω_* after the interaction length *L* in the crystal could be calculated as Equation (2),

(2) $$I_{3\omega }\;\left(L\right)=\frac{9\omega^{2}}{16c^{4}n_{\omega }^{3}n_{3\omega }\varepsilon_{0}^{2}}\;{\left|\chi_{eff}^{\left(3\right)}\right|}^{2}I_{\omega }^{3}\left(0\right)L^{2}\frac{sin^{2}\left(\frac{\Delta kL}{2}\right)}{{\left(\frac{\Delta kL}{2}\right)}^{2}}$$

where *n_ω_* and *n_3ω_* is the refractive index of the FW and TH wave. Equation (2) is applicable for CW‐laser light, but can be, for reasonably wide pulses, be approximately applied for pulsed lasers. Hence we assumed the equation to be applicable to our 300 fs pulsed laser source [42, 52]. If Δ*k* ≠ 0, there is a phase mismatch and the TH intensity would be oscillated with a period of Λ = 2*L_c_* (green line in Figure 1b), where *L_c_* = π/Δk is commonly referred to as the coherent length. Clearly, in order to obtain effective energy accumulation in THG process, the phase‐matching condition Δ*k* = 0 should be satisfied. At this time, the value of the *sinc*‐function is 1 and the intensity of the third‐harmonic wave would be proportional to the square of the propagation distance, as shown by the blue line. This phase‐matching condition requires that the refractive index *n_ω_* and *n_3ω_* are equivalent (The calculation details were given in the Supplementary information). In traditional nonlinear crystals, this phase‐matching requirement was usually realized by utilizing the optical birefringence, such as α‐BBO [42] and KTP crystal [53] cut along a specific angle to meet the condition $n_{\omega }^{o}=n_{3\omega }^{e}$. However, birefringent index‐matching in crystals requires strong optical anisotropy, hence all isotropic crystals are precluded.

Here we propose a phase modulation strategy by creating an ordered structure in isotropic crystals to improve THG efficiency. As shown in Figure 1b, an ordered/disordered periodic microstructure arrangement was introduced within the crystal to achieve phase‐compensation in regular intervals. The ordered regions represent the unprocessed crystal with a large χ^(3)^ coefficient, while in the disordered region, the χ^(3)^ nonlinearity was partially erased by laser direct‐writing. As a result, the FW light propagating in the ordered region normally performs cubic frequency conversion and the intensity of TH light was gradually improved. At the propagation length of *L_c_*, a certain π‐phase mismatch between FW and TH wave was generated. Then, in the disordered region where the lattice was damaged by laser processing, the backflow of energy—from TH to FW—was inhibited, resulting in only phase compensation. Clearly, if we set the periodic length of ordered and disordered regions as the coherent length (*L_m_* = *L_n_* = *L_c_* = π/Δk), the intensity of THG would be continuously enhanced by this phase modulation strategy (red line in Figure 1b). We refer to this artificial crystal with periodic χ^(3)^ distribution as a new type of ‘nonlinear photonic crystal’. A detailed theoretical analysis was given in Supplementary Materials (Figures S1–S3).

Compared to traditional birefringent phase‐matched crystals, this nonlinear photonic crystal allows efficient frequency tripling in its whole transparent region without the limitation of refractive index. So it can greatly enlarge the alternative χ^(3)^‐based nonlinear optical materials. In Figure 1c, using YAG as an example, we can see that the THG signal of the meta‐YAG is enhanced by six orders of magnitude than that of the unprocessed YAG crystal. The optical conversion in meta‐YAG increases to 4.5×10^−3^, which is comparable to those phase‐matched crystals and surpasses all nonlinear meta‐surfaces, epsilon‐near‐zero (ENZ) films, and semiconductor monolayers (Figure 1d). Therefore, the inscription of nonlinear refractive index gratings into the crystal structure is a powerful and widely applicable approach to increase the conversion efficiency of the THG process.

### 2.2 Fabrication and Characterization of Nonlinear Photonic Crystal

In general, the third‐order nonlinear coefficient χ^(3)^ is much smaller than χ^(2)^, so the possible THG effect was usually overlapped by strong SHG in the non‐centrosymmetric crystals. Therefore, the centrosymmetric yttrium aluminum garnet (YAG) crystal was selected in our experiment. YAG is an optically isotropic crystal with cubic symmetry. It has a wide optical transmittance (0.2–5 µm), high thermal conductivity (13.1 W/m∙K), high Mohs hardness (8.0–8.5), and good chemical stability. In previous studies, YAG crystals were typically used as laser host crystals. Since the first Nd:YAG laser reported in 1964 [54], rare‐earth doped YAG crystals have been widely utilized in solid‐state laser systems. However, to the best of our knowledge, YAG has never been considered a nonlinear optical crystal for more than sixty years, because it cannot compensate for the refractive dispersion. In addition, its Raman gain is very weak (∼ 0.1 cm/GW at 1.06 µm) [55] and is also not considered a good stimulated Raman crystal. Under Kleinman's symmetry, YAG has two independent third‐order nonlinear coefficients [56], $\chi_{11}^{\left(3\right)}$ and $\chi_{16}^{\left(3\right)}$ it has a large nonlinear refractive index *n*_2_ = 6.13×10^−16^ cm^2^/W at 1030 nm [57], corresponding to a cubic coefficient χ^(3)^ = 3.07×10^−19^ m^2^/V^2^, which is higher than KTP (8.05×10^−22^ m^2^/V^2^) [53] and α‐BBO (8.79×10^−23^ m^2^/V^2^) [42]. Therefore, YAG should be a potential candidate for frequency tripling using our phase modulation strategy.

**FIGURE 2:**
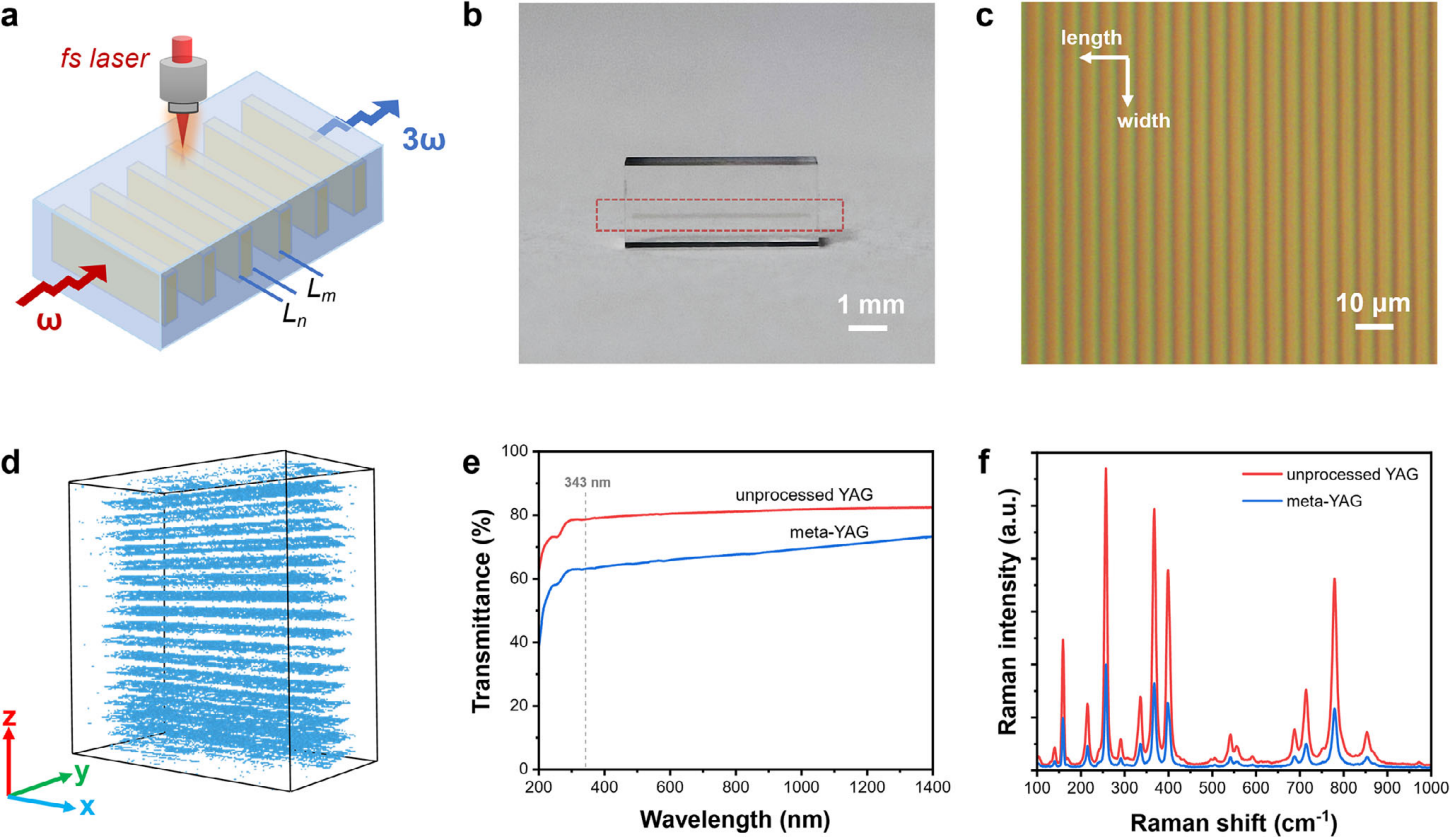
Femtosecond laser direct‐writing in meta‐YAG crystal. (a) Femtosecond writing setup for meta‐YAG. *L_m_* represents the length of the ordered region and L_n_ represents the disordered region of meta‐YAG. The laser writing direction is perpendicular to the propagation of FW light and TH light. (b) Photograph of meta‐YAG sample with the length of 5.37 mm, and the marked region by the red dotted box is laser writing region. Scale bar ∼ 1 mm. (c) Micrograph of meta‐YAG. The gray lines are disordered regions. Scale bar ∼ 10 µm. (d) 3D view of refractive index distribution in meta‐YAG. Blue regions represent the disordered areas. (e) Transmission spectra of unprocessed YAG and meta‐YAG written by laser energy of 0.2 µJ. (f) Raman spectra of unprocessed YAG and meta‐YAG. The excited wavelength is 532 nm.

In this work, a femtosecond direct‐writing method was applied to introduce microstructures into YAG crystal. The setup to inscribe the grating is shown in Figure 2a, and a Yb‐fiber laser with a central wavelength of 1030 nm was utilized. Figure 2b displayed the photograph of meta‐YAG crystal, where the marked position is the processed area visible to the naked eye. Using a microscope, it is observed that there are periodic microstructures in the meta‐YAG crystal (Figure 2c). Besides, using the refractive‐index imaging system to scan this meta‐YAG crystal, we can observe a clear periodic grating in the 3D image (The experimental setup was plotted in Figure S4). As shown in Figure 2d, the blue region represents the disordered region where the lattice structure was damaged by the high‐power femtosecond laser. It is observed that the microstructures in meta‐YAG crystal are very uniform and the ordered/disordered regions are alternately arranged.

Some optical characterizations were carried out to verify the optical properties of meta‐YAG crystal. Taking the meta‐YAG sample with a laser energy of 0.2 µJ, the optical transmittance at 343 nm decreased from 78.7% to 63.1% after the laser writing (Figure 2e), which could be attributed to additional scattering at the order/disorder interfaces. In addition, in Figure 2f, the intensity of Raman peak in the disordered region was significantly reduced, indicating that the crystal lattice of YAG was damaged after femtosecond laser writing and the lattice vibration was suppressed to a certain extent. As a result, it can be assumed that the χ^(3)^ coefficient is altered in the disordered regions as it is the case for SHG in a 3D LiNbO_3_ nonlinear photonic crystal [45]. In addition, we measured χ^(3)^ coefficients by the Z‐scan method (Figure S5), where the χ^(3)^ coefficients is reduced from 2.96×10^−19^ m^2^/V^2^ (YAG sample) to 1.56×10^−19^ m^2^/V^2^ (meta‐YAG sample). Therefore, the χ^(3)^ coefficients in the disordered region was changed and overall we confirmed a periodic χ^(3)^ distribution in the meta‐YAG crystal.

**FIGURE 3:**
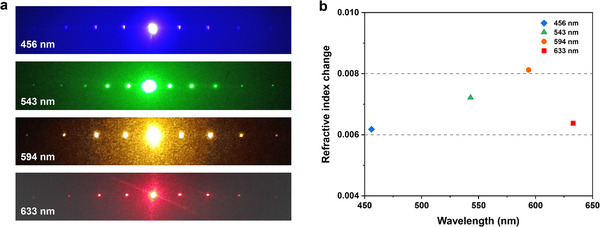
(a) The diffraction pattern of meta‐YAG crystal under 456, 543, 594 and 633 nm laser illumination. (b) The refractive index changes of the meta‐YAG crystal in order and disorder region.

In order to demonstrate the phase grating in meta‐YAG, we performed the optical diffraction under different wavelengths. The experimental setup was plotted in Figure S6 and diffraction patterns were collected on the optical screen. Figure 3a displayed the uniform 1D diffraction pattern at 456, 543, 594 and 633 nm, respectively. Based on the optical diffraction theory, the refractive‐index divergence Δn between the crystal region and the amorphous region was calculated (The detailed calculation methods were given in the Supplementary information). As shown in Figure 3b, the refractive index change Δn is very small and basically fluctuated in the range of 0.006‐0.008. Therefore, femtosecond laser‐writing does not change the refractive index of the crystal significantly and it is feasible to calculate the period of the phase‐grating based on the crystal dispersion.

**FIGURE 4:**
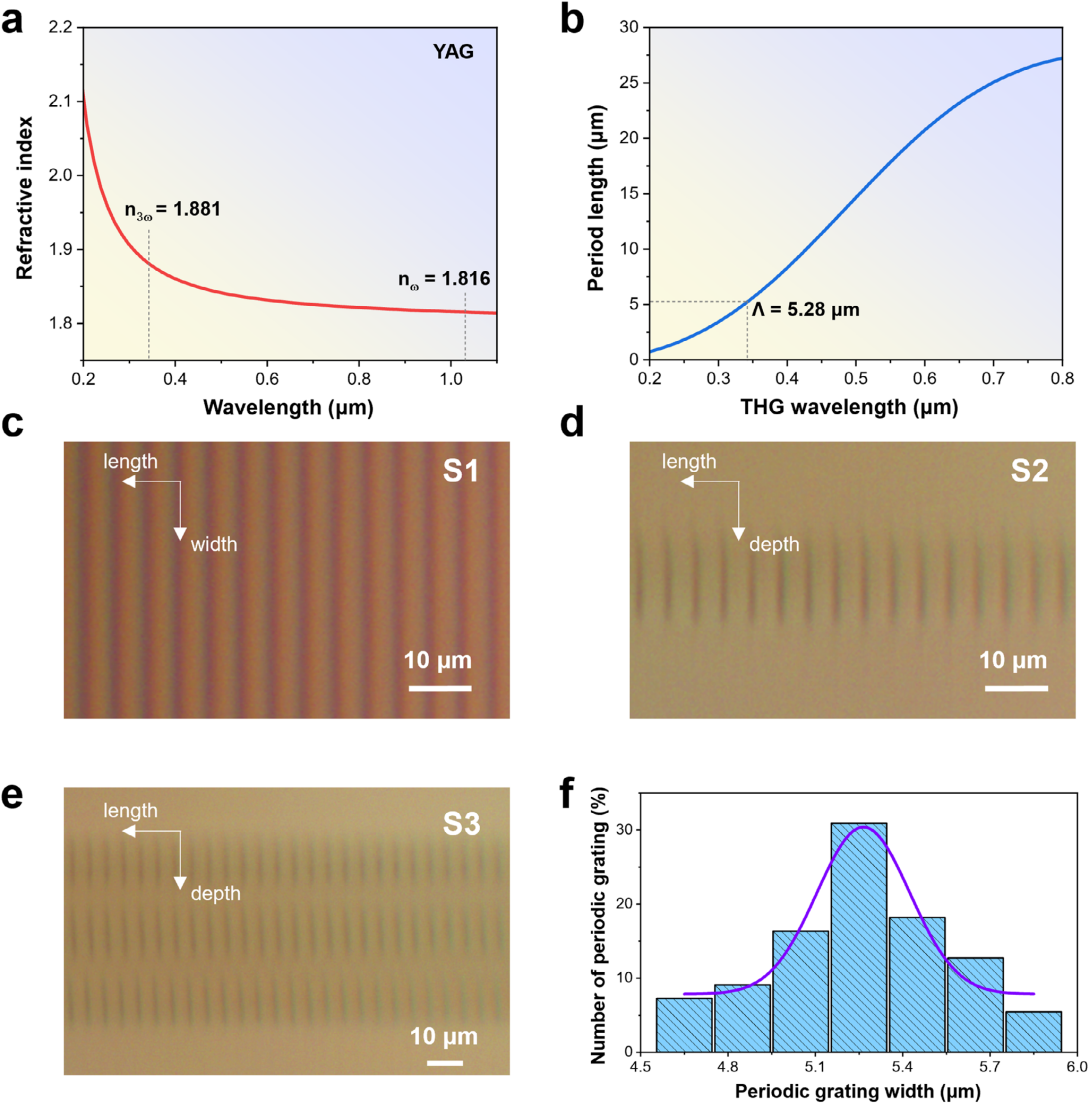
(a) Refractive index dispersion curve of YAG crystal [58]. (b) Calculated period length for THG meta‐YAG device. (c) Micrograph of S1 sample in the laser processing direction. Micrographs of (d) sample S2 and (e) sample S3 in the vertical track direction. (f) Grating period distribution statistics of sample S3. The period length is approximately ranged from 4.78 to 5.78 µm.

Next, we designed a meta‐YAG device for triple frequency‐conversion from 1030 nm to 343 nm. According to the phase modulation theory [15, 50], Equation (3) condition should be satisfied to improve the nonlinear frequency conversion,

(3) $$\Delta \varphi =\Delta \varphi_{m}+\Delta \;\varphi_{n}=\left(2a-1\right)\;\pi +\left(2b-1\right)\pi =2N\pi$$

where *a*, *b*, and *N* are integers. In order to get the highest conversion, we set the *a* = *b* = 1, that the phase modulation is Δφ  =  Δφ_*m*_ + Δ φ_*n*_ =  2π. Figure 4a shows the dispersion curve of YAG crystal, where the refractive index of *n_ω_* and *n_3ω_* is 1.816 and 1.881, respectively. Therefore, the phase difference of the frequency‐tripling was calculated as Equation (4),

(4) $$\Delta \;\varphi_{YAG}=\left(k_{3}-3k_{1}\right)\;z=2\pi \left(\frac{n_{3\omega }}{\lambda_{3\omega }}-\frac{3n_{\omega }}{\lambda_{\omega }}\right)z$$

Thus, the period of the phase grating is derived as $\Lambda_{YAG}=\frac{2\pi }{\Delta k}$ and the corresponding period of different tripling wavelength is shown in Figure 4b. For THG conversion of 1030 nm laser, the period length was determined to be 5.28 µm (The detailed calculation methods were given in the Supplementary information).

We designed three samples with different parameters, namely S1 (*N* = 650, *P* = 3), S2 (*N* = 1000, *P* = 1), and S3 (*N* = 1000, *P* = 3), where *N* represents the number of period cycles along the length‐direction and *P* is the number of layers in the track depth‐direction. A control group was set by changing the crystal size and the number of processing layers. The structural parameters of meta‐YAG were listed in Table S2. Figure 4c shows micrographs of the grating structure in the sample S1 along the vertical laser direct writing direction. Figure 4d,e show micrographs of sample S2 and S3 in the depth direction, respectively (Other micrographs of three samples are shown in Figure S7). Taking the influence factors such as mechanical vibration and movement deviation of the displacement table, the distribution of the grating period length in the sample S3 was calculated in Figure 4f. The central period is set as 5.28 µm and the period length of the phase grating ranges approximately from 4.78 to 5.78 µm. The period length error of the phase grating is ±0.5 µm and the corresponding phase fluctuation is ±9.47%. The duty cycle in meta‐YAG is 50%. The track depth for single layer is 20 µm.

The group velocity dispersion (GVD) of YAG crystal at 1030 nm is 66.7 fs^2^/mm. Theoretical calculations show that the effect of dispersion on the fundamental frequency pulse width is negligible for YAG crystal with a length of *L* = 5 mm at τ_0_ = 300 fs and λ = 1030 nm, with a dispersion variation of 300.02 fs. The distribution range of required period lengths is 5.12∼5.44 µm with the fundamental frequency spectrum width. Our processed meta‐YAG sample covers a period range of 4.78‐5.78 µm, which can effectively compensate the phase‐mismatch over the entire fundamental‐wave spectrum.

### 2.3 Direct Third‐Harmonic Generation in Meta‐YAG

**FIGURE 5:**
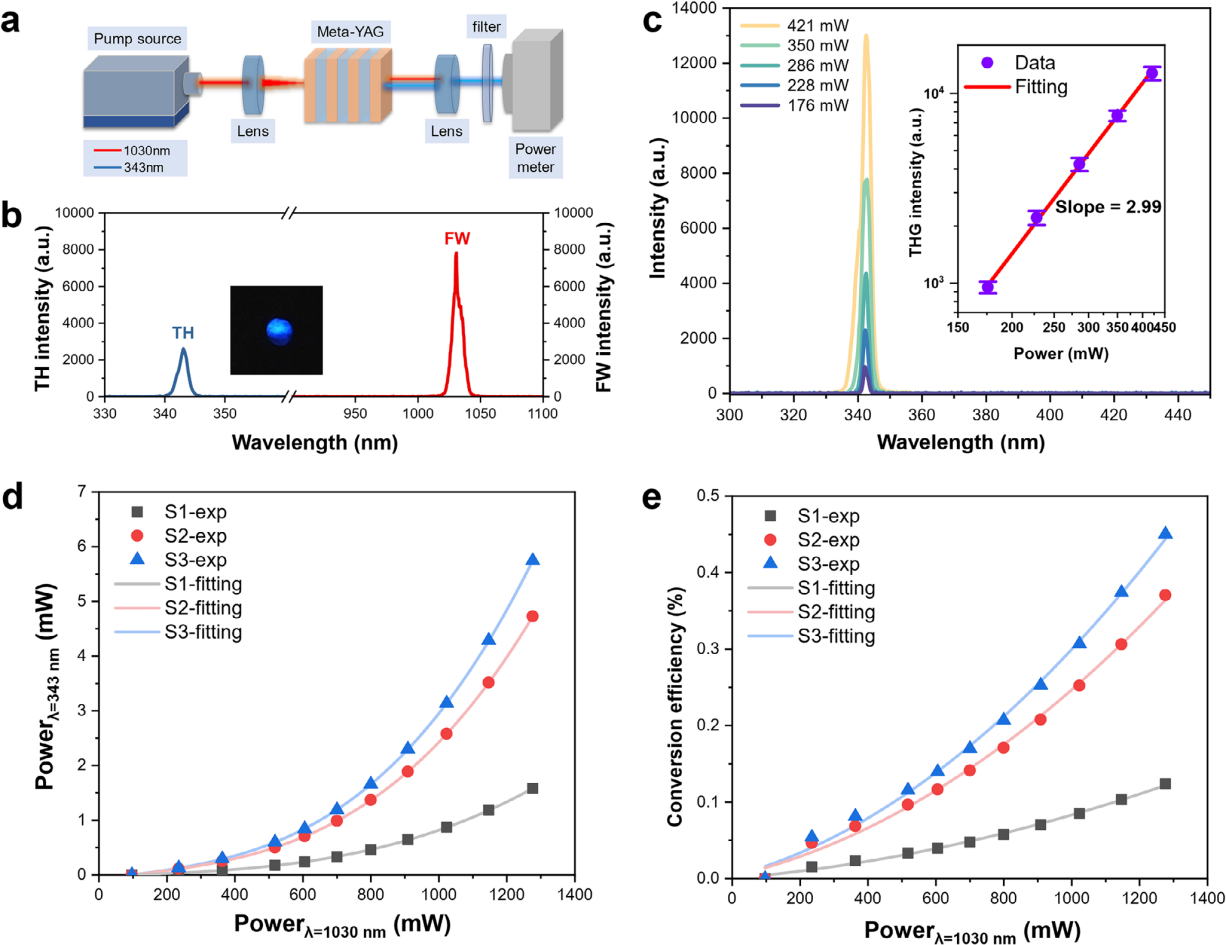
(a) Experimental setup for THG in meta‐YAG. (b) Laser spectrum of FW light at 1030 nm and TH signal at 343 nm. The insert graph is the fluorescence spot of 343 nm laser spot in UV region. (c) Power‐dependent THG spectra under different pump intensities. Inset shows the pump power dependence of THG intensity in logarithmic coordinate. (d) Output power at 343 nm in three different samples. (e) THG conversion efficiency in three different samples.

Figure 5a shows the experimental setup for THG in meta‐YAG device. The incident light was a femtosecond laser at 1030 nm with a pulse width of 300 fs. The spot diameter obtained through CCD measurement is determined to be 48 µm, which is comparable to the dimension of ordered structure in the meta‐YAG. A narrow‐band filter with T_oc_ = 80% at 343 nm was used to remove the residual FW light. Via a precise phase compensation from the additional microstructures, the THG frequency conversion could be greatly strengthened. As depicted in Figure 5b, a clear blue fluorescence spot was observed on the paper screen, corresponding to the THG signal at 343 nm. If we remove the incident light into the unprocessed YAG region, this fluorescent signal would disappear. The THG spectra of three samples S1, S2, and S3 and the photograph of 343 nm blue laser from the meta‐YAG sample were shown in Figure S8.

Moreover, we measured the evolution of spectral signal by changing the incident power of FW light. It was observed that the intensity of THG at 343 nm gradually increases with the increasing FW power, as shown in Figure 5c. The inset figure showed TH strength as a function of pump power with a perfect scaling law 𝐼 [3]. The fitted slope coefficient was 2.99, which confirms that the THG signal comes from the direct third‐harmonic generation process (*ω*+*ω*+*ω*→3*ω*), not a cascaded second‐order nonlinear process. Meanwhile, we measured the polarization of FW and TH light by a Glan prism, from which two beams hold the same polarization (Figure S9). This case identified that the frequency tripling process in meta‐YAG could be assigned to the (ooo‐o)‐type nonlinear conversion.

Figure 5d shows the input and output power of three different samples. Under the same pumping conditions of P_in_ = 1.27W, all three samples achieved the highest THG power output in the experiment. The maximum TH power at 343 nm in S1, S2, and S3 sample was 1.6, 4.7, and 5.8 mW, respectively. This THG power was much higher than that achieved in the index‐matched KTP (*P*_TH_ ∼ 0.68 µW at 531 nm) [53] and non‐phase‐matched diamond crystal (*P*_TH_ ∼ 0.15 mW at 426 nm) [52]. The S3 sample with 1000 period cycles and three layers of grating exhibited the highest TH power, which could be attributed to the precise phase modulation by the microstructures and the THG energy was accumulated to a higher level with the increase of the number of periods. As the number of grating layers increases, the size along the depth direction expands, which can cover a larger portion of the FW mode field and improve THG conversion efficiency.

The nonlinear efficiency of meta‐YAG was displayed in Figure 5e, where the S1 and S2 samples displayed a conversion efficiency of 1.2×10^−3^ and 3.7×10^−3^, respectively. In comparison, the S3 sample showed the highest conversion efficiency of 4.5×10^−3^ at the pump power of 1.27 W. If we deduct the absorption loss from filter and surface Fresnel loss, the actual THG efficiency in meta‐YAG was 0.68%, which is comparable to the birefringent phase‐matched THG crystal (η = 2%–3%) [42]. Notably, this micro‐structured design strategy could be applied to optical isotropic crystals, thus giving many opportunities to develop new nonlinear optical materials and devices, especially those large‐size crystals materials with high χ^(3)^ coefficients but no suitable birefringence. At this time, the input pump intensity is 2350 GW/cm^2^ and the normalized THG efficiency of meta‐YAG is 3.47×10^−13^/(GW/cm^2^). (The normalized efficiency comparison was shown in Figure S10). In addition, this high efficiency was better than those EZN materials and all‐dielectric structures, such as ITO film (*η* ∼ 7.05×10^−4^) [59] and silicon‐based meta‐surfaces (*η* ∼ 1.13×10^−5^) [41]. This result demonstrated the validity of phase modulation in the isotropic YAG crystal.

In addition, we also changed the repetition rate of the 1030 nm fundamental frequency light to investigate the nonlinear frequency conversion of meta‐YAG under different experimental conditions (Figure S11). It can be seen that the low repetition frequency leads to high peak power, as well as the improved THG power and conversion efficiency at the same pump power. Moreover, the power stability, beam quality of THG light, and THG signal change at various processing parameters, are shown in the Figures S12–S14.

### 2.4 Tunable Broadband THG in Meta‐YAG

Besides high efficiency, the wide spectral tuneability was also important in designing an entangled light source in quantum optics. In the index‐matched nonlinear crystals, the allowed THG bandwidth is usually small owing to the strict phase‐matching condition $n_{o}=n_{e}^{\theta }$ and the walk‐off angle is very large [33]. Therefore, only a narrow THG conversion could be achieved in a given crystal with a special cutting angle. At present, it is difficult to achieve broadband spectral tuning in the index‐matched crystals.

**FIGURE 6:**
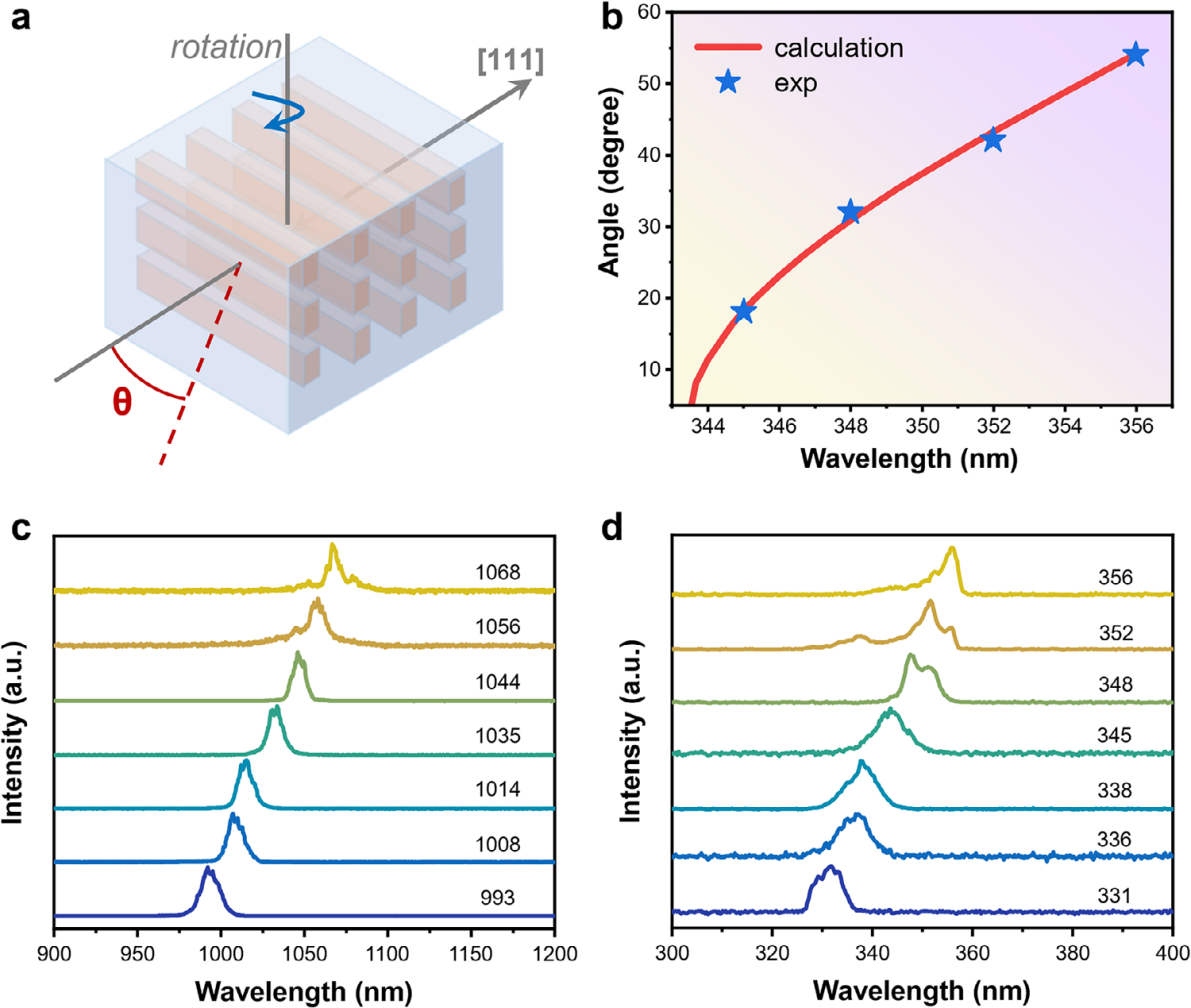
Angular tunable THG experiment in meta‐YAG. (a) Schematic diagram of angle tuning principle. (b) The relationship between the incident angle of the FW light and the output THG wavelength. When the angle is adjusted between 11.5°–54.2°, the obtained THG wavelength can cover the range of 343–356 nm. (c,d) FW and THG spectra for wide band tunable frequency conversion experiments.

In our designed meta‐YAG crystal, this problem could be solved by rotating the crystal. The effective periodic length of meta‐YAG could be continuously adjusted to satisfy the phase‐matching condition at different laser wavelengths. Figure 6a shows the schematic of angular tuning of THG experiments. The initial light propagation is along the (111) direction, and the meta‐YAG was rotated along the (01$\bar{1}$) direction. The rotation angle θ was set as the included angle between the wavevector of incident light and the YAG‐(111) direction. Figure 6b gives the corresponding relationship between rotation angle θ and THG wavelength. When the rotation angle changed from 11.5° to 54.2°, the effective period length was enlarged from 5.31 to 5.90 µm and the obtained THG wavelength could be tuned from 344 to 356 nm. The experimental results were well consistent with theoretical calculations. Moreover, from Figure 4f, there were some periodic length errors from laser direct‐writing, so that the period is not a fixed value but fluctuated in a certain range. As a result, this would introduce the phase fluctuation of random distribution in a certain interval, which is beneficial for broadband THG in meta‐YAG crystal.

Herein, we performed the THG experiment in the rotating meta‐YAG. Figures 6c,d show the spectra of FW and the related TH light. A wide THG conversion at 331‐356 nm was realized, which could be assigned to the synergistic contribution of angular tuning and random phase fluctuation. According to the angular phase regulation theory [60], the effective grating period under angle tuning is $\Lambda_{\mathrm{eff}}=\Lambda /\cos\theta \geq \Lambda$. Under this condition, the angular tuning range of 11.5°–54.2° maps to an effective period of the pump source spanning 5.28–5.9 µm, which in turn corresponds to the phase addition for the longer TH wavelength range of 343–356 nm. The theoretical design of the grating period is Λ = 5.28 µm, corresponding to the addition of π‐phase with THG wavelength of 343 nm at the normal incidence. Therefore, THG signal in 343–356 nm could be attributed to the angular phase regulation of periodic microstructures. For those short wavelengths at 331–343 nm, small phase grating periods are required to achieve π‐phase addition. Statistically, the whole grating period Λ = *L_m_* + *L_n_* was distributed in the range of 4.78–5.78 µm. Accordingly, the participation of phase fluctuations satisfies the phase regulation in the shortwave band, thus realizing the frequency tripling conversion at 331–343 nm. In previous report, Aizitiaili et al. have successfully shown the wideband spectral tuning in the non‐phase‐matched diamond [52], but their TH light linewidth was very large (∼15 nm). In contrast, the linewidth of THG obtained in meta‐YAG crystal was reduced to 3.68–7.09 nm, which should be favorable to improve the practicality and accuracy of multi‐wavelength frequency tripling. Therefore, nonlinear meta‐YAG would provide a new platform for χ^(3)^‐based integrated photonics, which maybe of equal importance to silicon‐based photonics.

## 3 Conclusion

In summary, we fabricated a meta‐YAG device by a microstructure strategy for boosting the third‐order nonlinear THG. An efficient frequency tripling was realized in this artificially designed YAG crystal, where the efficiency is enhanced to 10^6^ times that of the unprocessed YAG crystal, and the maximum THG efficiency of 4.5 × 10^−3^ was demonstrated. In addition, a tunable THG output from 331 to 356 nm was achieved, which indicated its great potential for broadband THG conversion. This method provides a feasible way for phase modulation in isotropic optical materials, which could be extended to many nonlinear crystals and glasses with large χ^(3)^ coefficients, e.g. diamond, Ba(NO_3_)_2_, and YVO_4_. In the future, THG power could be improved by selecting high‐performance materials, flexible 2D/3D microstructures, as well as the optimized thermal management technology, further verifying the validation of the strategy's feasibility and universality. Besides THG effect, this method could be applied in the efficient third‐order parametric down‐conversion [31], which makes intrinsic triple photon entanglement and creates quantum states beyond Gaussian statistics. Moreover, combining with solid‐state laser technology, some interesting self‐frequency conversion [61] and on‐chip laser sources could be designed, thus building a connection between laser and optical modulation. Finally, some nonlinear Moiré and twisted superlattice [62, 63, 64, 65] could be fabricated by two rotated meta‐YAG crystals, which gives intriguing properties, e.g. nondestructive detection of super‐resolution, in the third‐order nonlinear photonic crystals.

## 4 Experimental Section

### 4.1 Sample Preparation

YAG crystals were provided by Chengdu Dongjun Laser Co., Ltd. Pure YAG crystals were cut along the direction (111) with sizes of 2 × 2 × 3 mm^3^ and 2 × 2 × 5 mm^3^ (wide × thickness × length). The grating period of 5.28 µm was designed to produce the tripled 343 nm UV laser output in YAG crystal. Femtosecond laser direct‐writing was performed using a Yb‐fiber laser with a central wavelength of 1030 nm, a repetition frequency of 500 kHz and a pulse width of 200 fs. The laser light is focused into the YAG sample by a beam‐shaping system, including half‐wave plate, optical lens group, mirror, microscope objective (OptoSigma NIR50×, N.A. = 0.45) and other optical elements. The YAG sample was placed on a 3D precision displacement table with a writing speed of 10 mm/s. Taking the sample S3 with the best nonlinear frequency conversion effect as an example, its processing power is 100 mW, with peak power density is 16.4 TW/cm^2^. The single pulse energy is ∼0.2 µJ, and the line width of a single direct writing trace is ∼2 µm. Combining the repetition rate and laser scanning speed, an energy deposition rate parameter could be defined as $E_{d}=E\frac{F}{v}$, where *E_d_* is the energy deposition rate (µJ/µm), E is pulse energy, F is repetition rate of the laser, and v is the scanning speed. The calculated *E_d_* value during the processing of S3 sample is 10 µJ/µm [66].

Via controlling the parameters of the platform motion, the 1D photonic crystal structure with sub‐micron precision can be fabricated in meta‐YAG crystal. The distance between the phase grating and the crystal top‐surface is about 750 µm. The 5 mm and 3 mm crystals were arranged with 1000 and 650 grating cycles, respectively. The single‐layer structure is 20 µm in the depth direction, and the interval between each layer structure is 5 µm.

### 4.2 Sample Characterization

A 3D refractive index imaging system (Innofocus, nanoLABHoloview3D Ri (H3D)) was used to scan the sample and characterize the refractive index changes at 633nm in the processing area. During the characterization process, the 633nm helium neon laser was split into two coherent beams. One beam entered the CCD camera, while the other beam was focused by the objective lens and entered the sample slice from one side before being collected by the same CCD camera from the other side. By analyzing interference fringes of two coherent light collected by the CCD camera, refractive index changes in femtosecond‐laser‐induced tracks and the surrounding regions will be obtained [67].

The transmission spectra of unprocessed YAG and meta‐YAG at room temperature were measured with Shimazu UV2600i spectrophotometer in the wavelength range of 200–1200 nm with a data interval of 0.2 nm. The Raman spectra of the crystals were measured with LabRAM HR Evolution Raman spectrometer and the excitation laser wavelength was 532 nm. The integration time is 30 s. The diffraction pattern was collected through the crystal at the visible laser light of 633 nm (He‐Ne laser), 594 nm (He‐Ne laser), 543 nm (He‐Ne laser), and 456 nm (laser diode), respectively. The changed refractive index corresponding to the laser wavelength was measured by accurately measuring the power of the diffraction spots at different wavelengths.

### 4.3 THG Laser Experiment

A Yb‐fiber femtosecond laser at 1030 nm with a pulse width of 300 fs and a repetition frequency of 10–100 kHz, was utilized as the FW light in direct THG experiment. It was purchased from Tianjin BWT Femtosecond Laser Model (BFL‐1030‐10H). The maximum power was 10 W and the typical beam quality M^2^ factor was 1.4. The track length in the depth direction of the phase grating is *L_d_* = 20 and 70 µm, so the FW beam is focused into the meta‐YAG crystal by *f* = 50 mm focusing mirror to ensure that all fundamental‐wave energy can pass through the phase grating. The obtained TH signal is collected by a power meter after a narrow‐band filter (T_oc_ = 80% at 343 nm). The tunable THG experiments were performed by an ORPHEUS collinear optical parametric amplifier (OPA) system. A broadband laser from 890 to 1100 nm (output power ∼ 150–300 mW, pulse energy ∼ 3–7 µJ, repetition rate ∼ 100 kHz, and pulse duration ∼ 250 fs) was utilized as a FW light to obtain the broadband THG from 331 to 356 nm.

## Author contributions

F.L. and H.H.Y. conceived and supervised this project. X.T.G. and Q.L.H. performed optical experiments and wrote the manuscript. B.Z. built the laser‐writing platform. D.Z.L and H.J.Z. provided helpful suggestions for the design of meta‐YAG. All authors contributed to the discussion and preparation of the manuscript.

## Conflicts of Interest

The authors declare no conflict of interest.

## Supporting information




**Supporting File**: advs74379‐sup‐0001‐SuppMat.docx

## Data Availability

The data that support the findings of this study are available from the corresponding author upon reasonable request.
