# Supplementary material for: High‐Order Nonlinear Photonic Crystal with Ordered Structures for Giant Enhancement of Frequency Tripling
Source: Adv Sci (Weinh). 2026 Feb 11;13(23):e24069. doi: 10.1002/advs.202524069 (PMC13104140; doi:10.1002/advs.202524069)
Supplement: Supplementary file 1 — Supporting File: advs74379‐sup‐0001‐SuppMat.docx [file ADVS-13-e24069-s001.docx]

**Supplementary Information for**

**High-order nonlinear photonic crystal with ordered structures for** **giant enhancement of frequency tripling**

Xiaotian Guo^1^, Qiaoling Han^1^, Fei Liang^1,*^, Bin Zhang^2^, Dazhi Lu^1^, Haohai Yu^1,*^, Huaijin Zhang^1^

^1^ State Key Laboratory of Crystal Materials and Institute of Crystal Materials, Shandong University, Jinan 250100, China

^2^ School of Physics, Shandong University, Jinan 250100, China

Corresponding authors

Email:

* liangfei@sdu.edu.cn (F. Liang)

* haohaiyu@sdu.edu.cn (H. H. Yu)

### 1. Theoretical derivation of nonlinear THG process

According to the basic theory of nonlinear frequency conversion^[1-3]^, the polarization intensity of the TH is

$$\begin{aligned} P_{3\omega}^{\left( 3 \right)}=\varepsilon_{0}\chi^{\left( 3 \right)}\left( 3\omega;\omega,\omega,\omega\right)E_{\omega}^{\left( 3 \right)}\#\left( 1 \right) \end{aligned}$$

Here, ε_0_ represents the dielectric constant of the vacuum. For the tripling effect, the approximate equation for the slow amplitude of the plane wave propagating in the Z direction is^[4]^

$$\begin{aligned} \frac{{dE}_{3\omega}\left( z \right)}{dz}=\frac{3i\omega\mu_{0}\varepsilon_{0}c}{2n_{3\omega}}\chi^{(3)}E_{\omega}^{3}e^{i\Delta kz}\#\left( 2 \right) \end{aligned}$$

Where μ_0_ is the vacuum permeability, n_3ω_ is the refractive index of the TH transmission in the medium, i and c are the complex unit and the speed of light in vacuum, respectively. In the formula, the wave vector difference $\Delta k$ is: $\Delta k=k_{3\omega}-3k_{\omega}$.

Based on small signal approximation, which says that there’s no attenuation to be considered on fundamental frequency light within the action length L, that is, $E(z,\omega)=E(0,\omega)$. Under the plane wave approximation, substituted the relationship between light intensity and amplitude, the expression of the TH intensity I_3ω_ after the interaction length L in the crystal is as follows^[5]^:

$$\begin{aligned} I_{3\omega}\left( L \right)=\frac{{9\omega}^{2}}{16c^{4}n_{\omega}^{3}n_{3\omega}\varepsilon_{0}^{2}}\left| \chi^{(3)} \right|^{2}I_{\omega}^{3}\left( 0 \right)L^{2}\frac{{sin}^{2}\left( \frac{\Delta kL}{2} \right)}{\left( \frac{\Delta kL}{2} \right)^{2}}\#\left( 3 \right) \end{aligned}$$

From the properties of sinc function, it is not difficult to find that in order to obtain efficient nonlinear frequency conversion, the phase matching condition of $\Delta k=0$ must be satisfied.

In the absence of crystal absorption and scattering of light and other conditions, from the fundamental frequency light to triple frequency light this system should comply with the conservation of energy and momentum, according to the conservation of energy, there are natural:

$$\begin{aligned} \omega_{1}+\omega_{1}+\omega_{1}=\omega_{3}=3\omega_{1}\#\left( 4 \right) \end{aligned}$$

When the phase difference is 0, the system has no momentum loss, and the momentum relationship can be expressed as

$$\begin{aligned} k_{3}=3k_{1}\#\left( 5 \right) \end{aligned}$$

By definition, the wave vector *k* is

$$\begin{aligned} k=\frac{n}{c}\omega\boldsymbol{k}\#\left( 6 \right) \end{aligned}$$

where $n=n(\omega)$ is the refractive index of the crystal with frequency ω, and $\boldsymbol{k}$ is the unit wave vector. Substitute equation (6) into equation (5) to get:

$$\begin{aligned} n_{3}\left( \omega_{3} \right)\omega_{3}=3n_{1}\left( \omega_{1} \right)\omega_{1}\#\left( 7 \right) \end{aligned}$$

Considering equation (4), the above equation can be rewritten as

$$\begin{aligned} n_{3}\left( \omega_{3} \right)=n_{1}\left( \omega_{1} \right)\#\left( 8 \right) \end{aligned}$$

or simply expressed as

$$\begin{aligned} n_{3\omega}=n_{\omega}\#\left( 9 \right) \end{aligned}$$

In order to obtain effective energy accumulation in nonlinear frequency conversion, condition $n_{3\omega}=n_{\omega}$ must be satisfied. The traditional phase-matching method requires crystals to have anisotropy. This is contrary to the choice of requiring crystal isotropy in order to take advantage of larger third-order nonlinear coefficients in the medium. Therefore, we consider how to realize phase regulation in isotropic crystals to achieve phase matching, so as to achieve effective direct THG.

### 2. Principle of Phase Modulation in Microstructure Strategy

Owing to the translational symmetry of the crystal lattice, the physical properties at an arbitrary lattice site $\boldsymbol{r}$are identical to those at the site $\boldsymbol{R}=r+l_{1}\boldsymbol{\alpha}_{\mathbf{1}}+l_{2}\boldsymbol{\alpha}_{\mathbf{2}}+l_{3}\boldsymbol{\alpha}_{\mathbf{3}}$. Herein, $\boldsymbol{R}$is defined as an arbitrary lattice periodic vector, while $\boldsymbol{R}$and $\boldsymbol{l}_{\boldsymbol{n}}$(n=1,2,3) denote the lattice basis vectors and arbitrary sets of integers, respectively. Therefore, the lattice potential field $V\left( \boldsymbol{r} \right)$at different lattice sites satisfies the following relationship:

$$V\left( \boldsymbol{r} \right)=V\left( \boldsymbol{r}+l_{1}\boldsymbol{\alpha}_{\mathbf{1}}+l_{2}\boldsymbol{\alpha}_{\mathbf{2}}+l_{3}\boldsymbol{\alpha}_{\mathbf{3}} \right)$$

This expression manifests the periodic characteristic of the potential field function. According to Bloch's theorem, in such a periodic lattice potential field, the moving particles inside the crystal are modulated by the lattice periodicity, and the solution $\Psi$ to the wave equation can be expressed as:

$$\Psi\left( \boldsymbol{r}+\boldsymbol{R} \right)=e^{i\boldsymbol{k}\cdot\boldsymbol{R}}\Psi\left( \boldsymbol{r} \right)$$

It can be seen from this formula that after the particle is translated by the lattice vector $\boldsymbol{R}$, a phase factor $e^{i\boldsymbol{k}\cdot\boldsymbol{R}}$is introduced compared with the phase of the particle before translation. When the phase factor satisfies $\boldsymbol{k}\cdot\boldsymbol{R}=2N\pi$(N is an integer), the equation is simplified to $\Psi\left( \boldsymbol{r}+\boldsymbol{R} \right)=\Psi\left( \boldsymbol{r} \right)$. In this case, the particle still maintains its original physical properties after being translated by the lattice periodicity.

In nonlinear optics, the wave function of a plane wave also exhibits a similar periodic property; the phase of the coupled wave remains unchanged after a periodic translation of $2N\pi$, which can be expressed as:

$$\Psi\left( \varphi\right)=\Psi\left( \varphi+2N\pi\right)$$

This phase relationship provides a novel insight for considering the phase matching condition. Specifically, the strict phase matching condition $\Delta\varphi=0$can be relaxed to $\Delta\varphi=2N\pi.$ Instead of relying on the stringent phase matching condition, we realize the periodic phase modulation by introducing microstructures via femtosecond laser direct-writing. Meanwhile, the additional phase modulation within a single period can prevent the oscillation of energy between coupled waves and thus avoid the failure of energy accumulation within one period.

The fundamental theory of this phase modulation strategy based on artificial microstructures lies in the periodic characteristic of the coupled wave phase. As illustrated in the **Figure S1**, the period length of the introduced phase grating is $\Lambda=L_{m}+L_{n}$, where $L_{m}$ and $L_{n}$represent the lengths of the ordered lattice region and the disordered region, respectively, and $\chi^{\left( 3 \right)}$denotes the nonlinear coefficient of the ordered region.

Through the periodic disruption of the crystal structure, we artificially introduce a periodic array of ordered/disordered phase grating microstructures inside the crystal. Within a single period corresponding to a phase variation of 2π, the phase difference between the coupled waves in the ordered region is less than π, which ensures that the energy is constantly transferred from the fundamental frequency light to the third harmonic wave, thus enabling efficient nonlinear frequency conversion. If the length of the ordered region is adjusted to be equal to one coherence length L_c_, the maximum phase difference $\Delta\varphi_{m}=\pi$can be achieved at the interface between the ordered and disordered regions, which ensures that the energy continuously flows to the third harmonic without backflow, and the energy accumulation reaches the maximum at this moment. When the light propagates into the disordered region, the phase difference in this region is also adjusted to $\Delta\varphi_{n}=\pi$by controlling the length of the disordered region, which exactly compensates for the phase difference generated in the last ordered region. In the disordered region, the nonlinear coefficient $\chi^{\left( 3 \right)}$is reduced (altered), thus hindering the backflow of third-harmonic wave energy. After the light propagates through one structural period, the total phase difference of the coupled waves reaches$\Delta\varphi=\Delta\varphi_{m}+\Delta\varphi_{n}=\pi+\pi=2\pi$, accompanied by the efficient accumulation of energy. This process is repeated in the subsequent identical periodic structures, leading to a gradual enhancement of the harmonic energy and thus realizing high THG conversion efficiency.


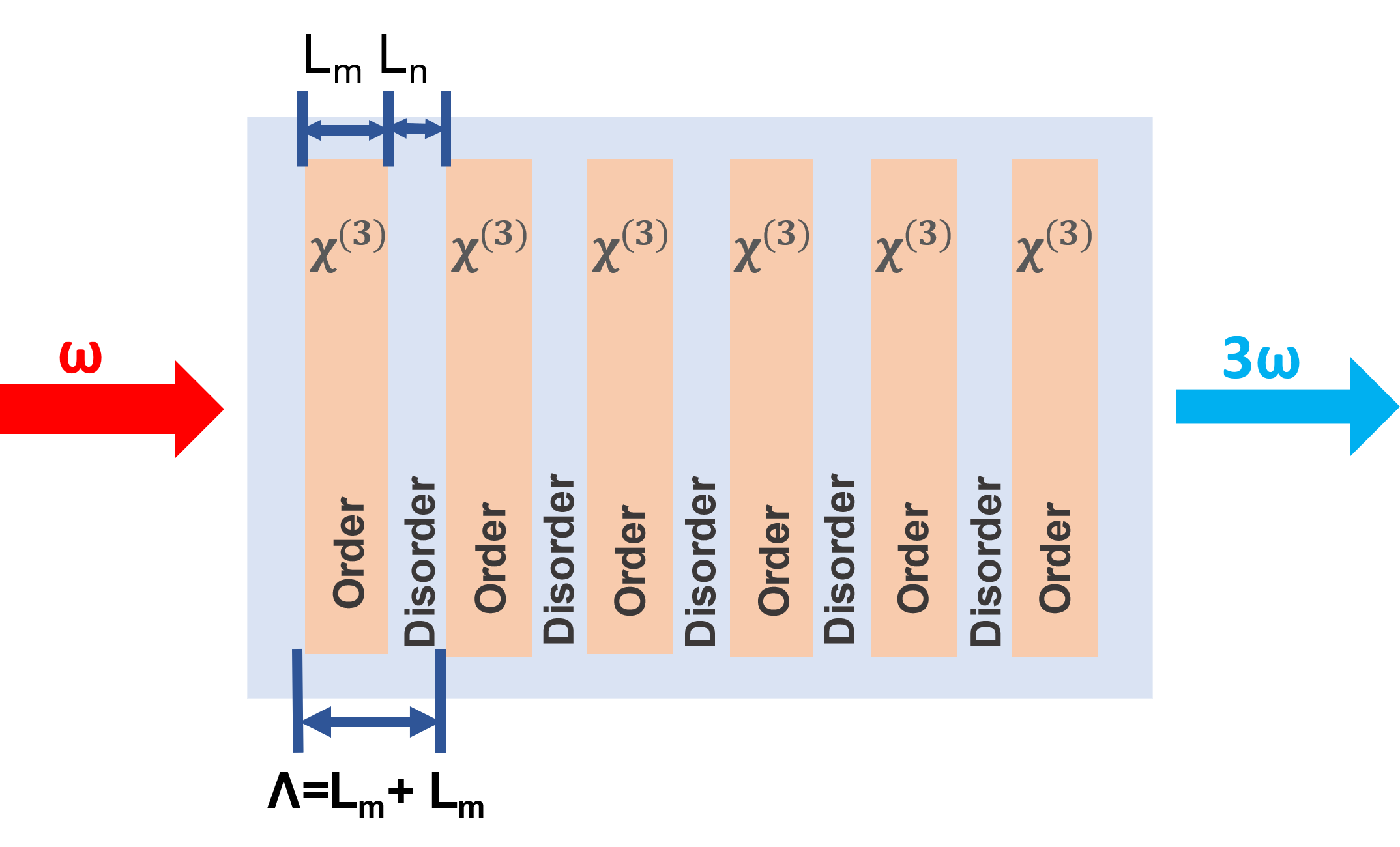


**Supplementary Figure S1.** Schematic diagram of periodic phase grating structure.

### 3. The influence of duty cycle on THG efficiency

As can be seen from the fundamental principle of the microstructure strategy, taking the direct THG process as an example, its electric field expression is given by:

$$\frac{dE_{3\omega}\left( z \right)}{dz}=\frac{3i\omega d\left( z \right)}{2n_{3\omega}\left( z \right)c}E_{\omega}^{3}\left( z \right)e^{-i\Delta kz}$$

Where $n_{3\omega}\left( z \right)$and $d\left( z \right)$represent the third-harmonic refractive index and the effective third-order nonlinear coefficient dependent on the propagation distance z, respectively. According to the above expression, considering the two influencing factors, namely the third-harmonic refractive index (which differs slightly between the ordered and disordered regions due to lattice damage induced by femtosecond laser processing) and the third-order nonlinear coefficient (where d_eff_ is adopted for the ordered region and approximated to 0 for the disordered region to simplify calculations), their periodic distribution $\frac{d\left( z \right)}{n_{3\omega}\left( z \right)}$can be expressed in the form of Fourier transform:

$$\frac{d\left( z \right)}{n_{2\omega}\left( z \right)}=\frac{a_{ij}}{n_{2\omega}}f_{THG}\left( z \right)$$

where the periodic function $f_{THG}\left( z \right)$has the form:

$$f_{THG}\left( z \right)=C_{0}+\sum_{m\neq0}^{+\infty} C_{m}e^{iG_{m}z}$$

In this formula, the m order Fourier coefficient is expressed as:

$$C_{m}=\frac{1}{\Lambda}\int_{-\frac{\Lambda}{2}}^{\frac{\Lambda}{2}} f\left( z \right)e^{iG_{m}z}=\left\{ \begin{aligned} D \#m=0 \\ \frac{1}{m\pi}\sin\left( Dm\pi\right)\#m\neq0 \end{aligned} \right.$$

It can be deduced that the corresponding grating period length is $\Lambda=2m\frac{\lambda_{\omega}}{6\left( n_{3\omega}-n_{\omega} \right)}$, while the duty cycle of the ordered lattice region in the phase grating structure is defined as $D=\frac{L_{m}}{L_{m}+L_{n}}=\frac{L_{m}}{\Lambda}$.

Based on this theoretical analysis, it can be concluded that duty cycle is an important factor to determine the conversion efficiency in meta-YAG crystal. As shown in **Figure S2**, we present three cases of phase modulation, namely $\Delta\varphi_{m}=\Delta\varphi_{n}=\pi$, $\frac{1}{3}\Delta\varphi_{m}=\Delta\varphi_{n}=\pi$ and $\Delta\varphi_{m}=\Delta\varphi_{n}=3\pi$. It can be observed that with the increase of phase, all the above modulation modes can realize effective energy accumulation. However, with the same propagation length, the modulation case of $\Delta\varphi_{m}=\Delta\varphi_{n}=\pi$ (duty cycle=50%) achieves the highest conversion efficiency.

In experiments, we fabricated three meta-YAG samples with *L_m_* : *L_n_*=1, *L_m_* : *L_n_*=3, and *L_m_* : *L_n_*=5, whose periods are 5.28 μm, 10.56 μm and 15.84 μm, respectively. With same experimental setups (same pump light and same crystal length), the output THG signals collected by the spectrometer verifies that the sample-1 (with *L_m_*:*L_n_*=1) shows the maximum THG intensity (**Figure S3**). Therefore, the THG frequency conversion effect is optimal when a=b=1.


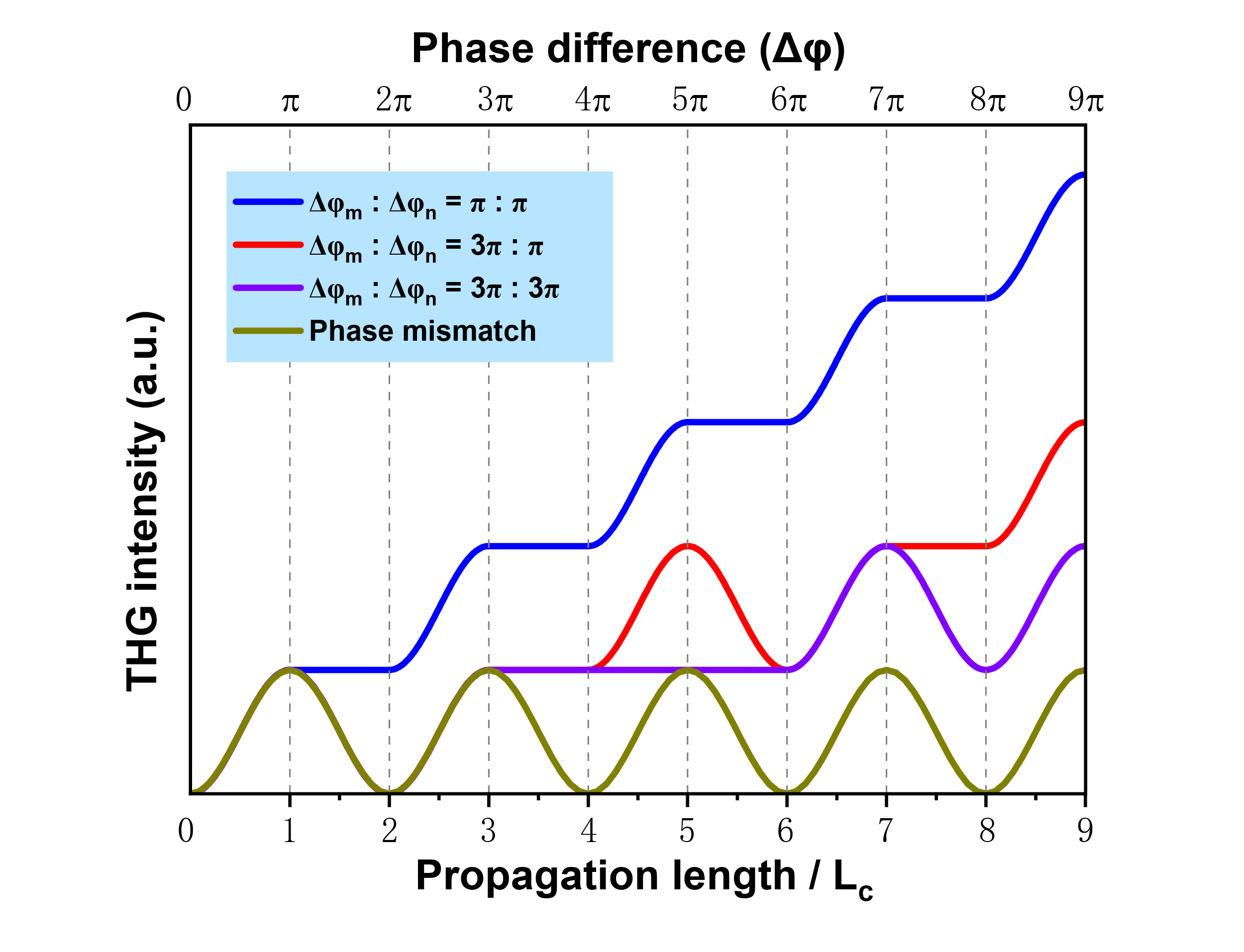


**Supplementary Figure S2.** Intensity accumulation of third harmonics under different phase addition conditions


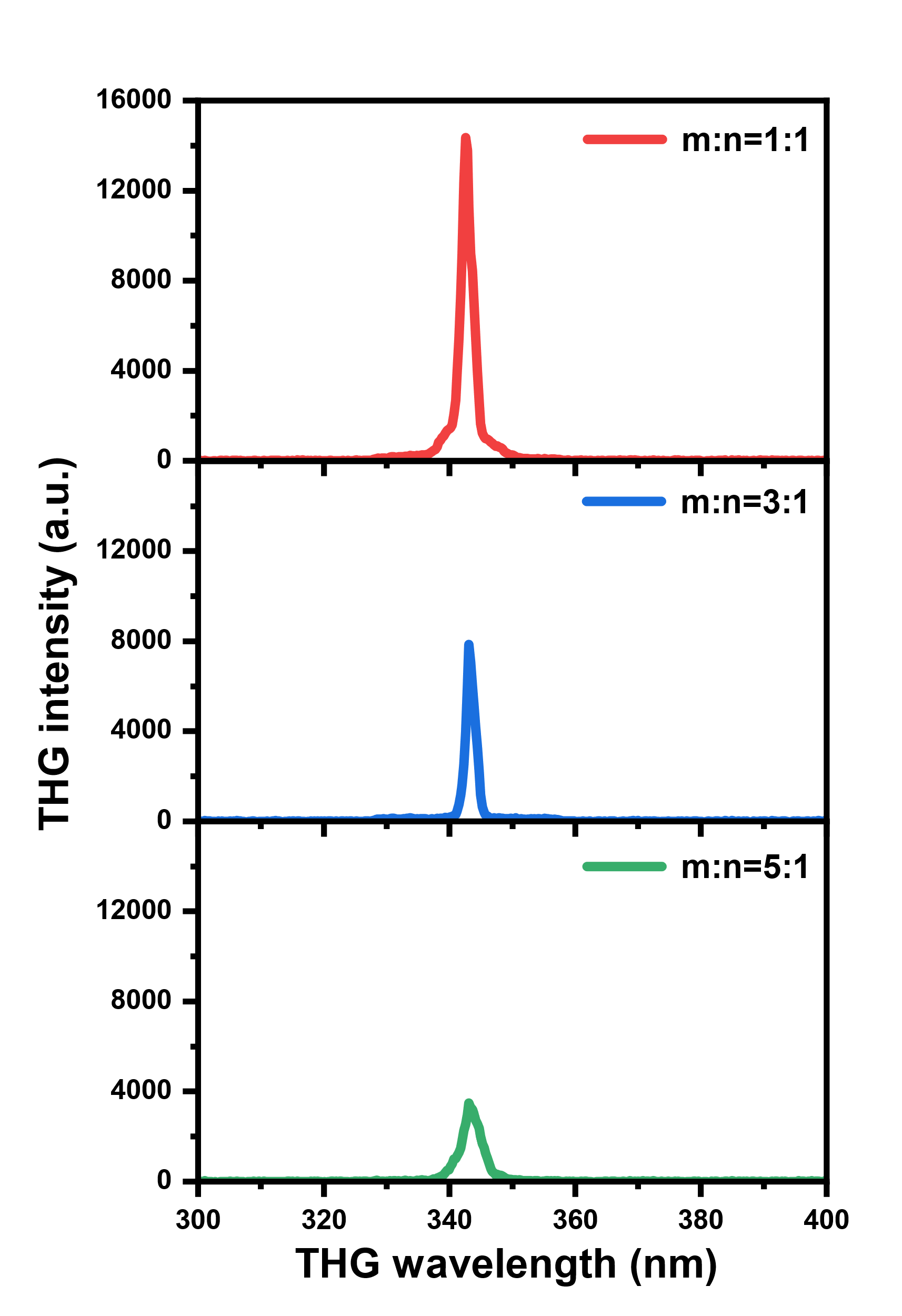


**Supplementary Figure S3.** Comparison of THG intensities of meta-YAGs with different duty cycles.

### 4. Calculation of refractive index change $\boldsymbol{\Delta n}$of nonlinear photonic crystal

Diffraction occurs when the light is transmitted in the phase grating, and diffraction patterns are formed when the laser of different wavelengths passes through the grating structure in the nonlinear photonic crystal and falls on the optical screen, as shown in **Supplementary Figure S1**. In order to obtain the periodic change of refractive index caused by periodic lattice destruction, we measure the efficiency of the series of spots with different diffraction levels according to Bragg diffraction theory. The phase difference resulting from a change in refractive index can be expressed as,

$$\begin{aligned} \Delta\varphi=\frac{2\pi\Delta nh}{\lambda}\#\left( 10 \right) \end{aligned}$$

Where $\Delta n$ represents the change of refractive index; h is the thickness of the volume grating. For rectangular gratings, the efficiency expression for each diffraction stage is

$$\begin{aligned} \left\{ \begin{aligned} \eta_{0}=1=2D(1-D)(1-cos\Delta\varphi) \\ \eta_{m}=\frac{1}{m^{2}n^{2}}\left( 1-cos2m\pi D \right)\left( 1-cos\Delta\varphi\right), m>0 \end{aligned} \right.\#\left( 11 \right) \end{aligned}$$

$\eta_{0}$and $\eta_{m}$ (m>0) are the diffraction efficiency of different orders, respectively, and D is the duty cycle of the phase grating. By substituting the measured data into equations (10) and (11), the change of refractive index at different wavelengths can be calculated. The refractive index difference of meta-YAG crystal at 456 nm, 543 nm, 594 nm and 633 nm fluctuates basically in the range of 0.006~0.008 in the visible region. Therefore, femtosecond laser-writing does not change the refractive index of the crystal significantly and it is feasible to calculate the period of the phase grating based the crystal dispersion.

### 5. The calculated period length of the meta-YAG crystal

According to the basic properties of the artificial microstructure theory, the ordered part $L_{m}$ for efficient nonlinear frequency conversion should satisfy the coupled wave phase relationship as follows:

$$\begin{aligned} \Delta\varphi_{m}=\Delta{kL}_{m}=\left( 2a-1 \right)\pi\#(12) \end{aligned}$$

However, there won’t exist energy exchange in the disordered region $L_{n}$, only the phase difference is provided:

$$\begin{aligned} \Delta\varphi_{n}=\Delta{kL}_{n}=\left( 2b-1 \right)\pi\#(13) \end{aligned}$$

In this way, the phase addition regulation is carried out through the disordered region, so as to make up for the phase mismatch caused by the frequency conversion in the ordered structure, and meet the conditions for adding periodic phase:

$$\begin{aligned} \Delta\varphi=\Delta\varphi_{m}+\Delta\varphi_{n}=\left( 2a-1 \right)\pi+\left( 2b-1 \right)\pi=2N\pi\#(14) \end{aligned}$$

Where a, b, and N are integers. Only when equations (12), (13) and (14) are satisfied at the same time can efficient frequency conversion be achieved.

Considering the realization of artificial microstructure phase adjust condition $\Delta\varphi=\Delta\varphi_{m}+\Delta\varphi_{n}=2\pi$, the phase difference of the frequency tripling process can be calculated as follows:

$$\begin{aligned} \Delta\varphi_{YAG}=\left( k_{3}-3k_{1} \right)z=2\pi\left( \frac{n_{3\omega}}{\lambda_{3\omega}}-\frac{{3n}_{\omega}}{\lambda_{\omega}} \right)z\#(15) \end{aligned}$$

Thus, the period of the phase grating is obtained as $\Lambda_{YAG}=\frac{2\pi}{\Delta k}$. In order to obtained the TH process of 343 nm laser in the ultraviolet band. The Sellmeier equation^[6]^ used in the calculation is shown in the Eq (16),

$$\begin{aligned} n^{2}=1.882+\frac{1.404\lambda^{2}}{\lambda^{2}-{0.1338}^{2}}-0.0137\lambda^{2}\#\left( 16 \right) \end{aligned}$$

The processing period was determined to be 5.28 μm according to the refractive index of YAG crystal.

### 6. The calculated frequency dispersion in THG process in meta-YAG crystal

During the THG process, femtosecond lasers will cause changes in pulse width. After the ultra short pulse passes through the dispersive element, the pulse shape will change. The dispersion provided by the dispersion element can be expanded by Taylor expansion, with the second-order dispersion β_2_ having the greatest impact on the pulse.

After introducing second-order dispersion into Gaussian pulses with Fourier limit width, the pulse is broadened, and the wideband of the broadened pulse is:

$$\begin{aligned} \tau=\tau_{0}\sqrt{1+\left( 4\ln2\frac{\beta_{2}}{\tau_{0}^{2}} \right)^{2}} \end{aligned}$$

Where $\tau_{0}$ represents the pulse width of the incident pulse (fs), β_2_ represents the dispersion of the medium (fs^2^), and $\tau$ represents the pulse width of the output pulse.

The medium dispersion β_2_ in the equation is defined by group velocity dispersion (GVD). To estimate the amount of dispersion introduced by a material with a length of L, we obtained the GVD parameters related to the second-order dispersion under 1030nm excitation of YAG by,

$$\begin{aligned} GVD=\frac{\lambda^{3}}{2\pi c^{2}}\left( \frac{d^{2}n}{d\lambda^{2}} \right) \end{aligned}$$

$$\begin{aligned} \beta_{2}=GVD\times L \end{aligned}$$

The group velocity dispersion of YAG crystal under 1030 nm pump can be found in ref, and GVD=66.7 fs^2^/mm can be obtained. Theoretical calculations show that the effect of dispersion on the fundamental frequency pulse width is negligible for a YAG crystal with a length of *L*=5 mm at $\tau_{0}$=300 fs and λ=1030 nm, with a dispersion variation of only 300.02 fs. As a result, the distribution range of Δk induced by dispersion is 5.12-5.44 μm under the fundamental frequency spectrum width. In comparison, our fabricated meta-YAG sample has a period range of 4.78-5.78 μm ($\Lambda=\frac{2\pi}{\Delta k}$), which can effectively utilize the wavelength range covered by the femtosecond fundamental light.

In addition, the optical dispersion for peak power is also negligible. Taking a pump power of 1.27W as an example, for a femtosecond laser with a pulse width of 300 fs and a repetition rate of 100 kHz, the peak power is 4.23333×10^7^. If the pulse width increased to 300.02 fs, the corresponding peak power is 4.23305×10^7^, with a variation rate of only 0.0067%. Therefore, GVD’s effect could be negligible in our experimental parameters.

### 7. Additional figures and tables


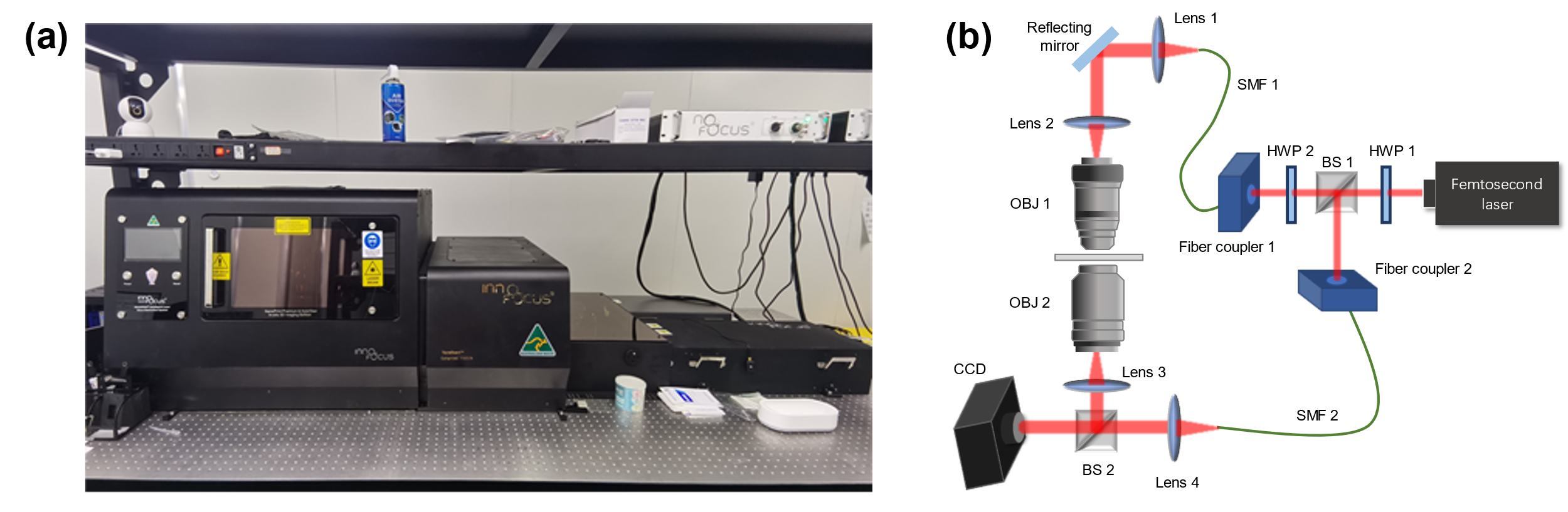


**Supplementary Figure S4.** a) Experimental setup and b) principle diagram of 3D refractive index imaging.


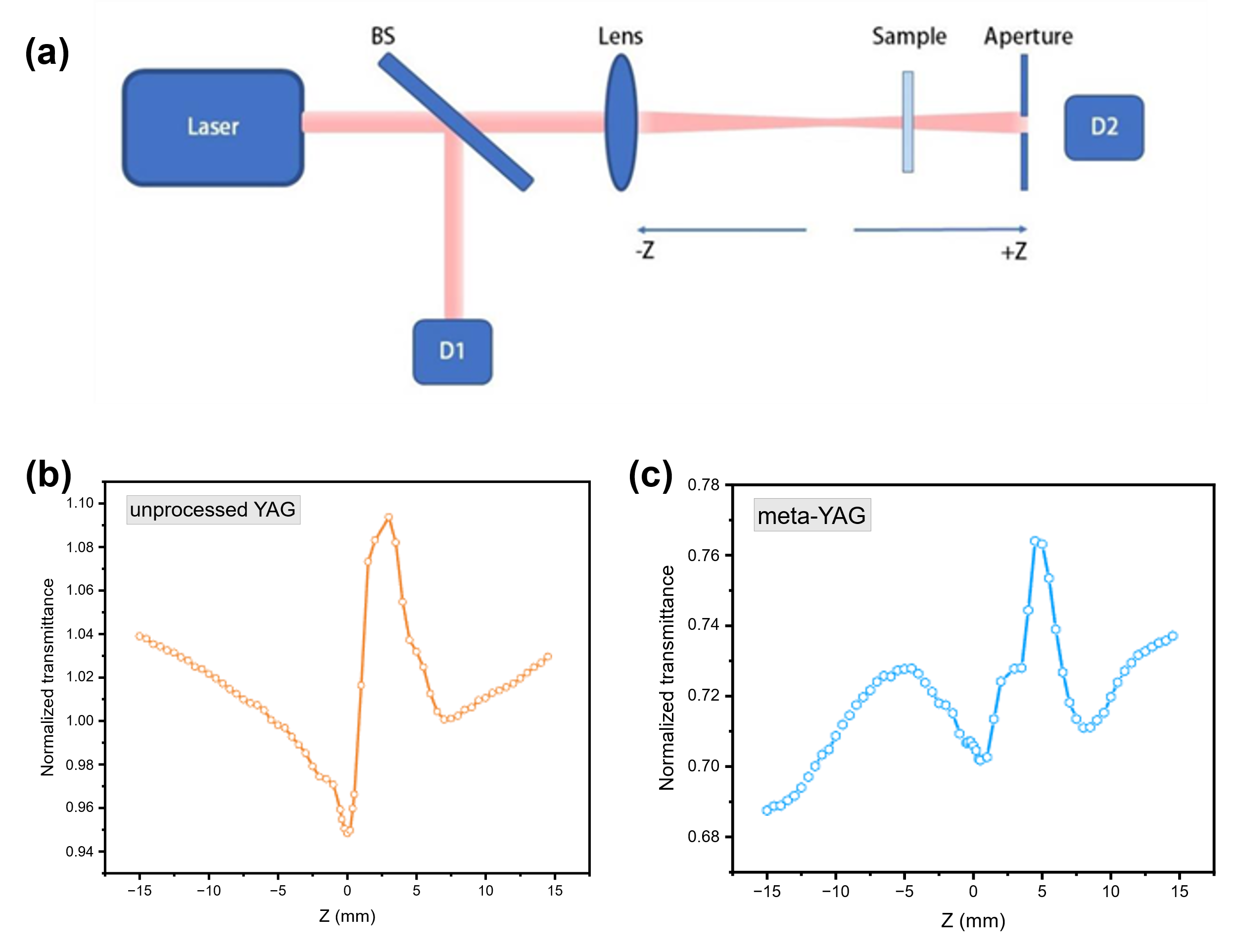


**Supplementary Figure S5.** (a) Experimental setup for Z-scan test. (b, c) Z-scan results for YAG and meta-YAG.

In order to verify the change of χ^(3)^ in the disordered regions, we provide a *Z*-scan result in the revision, which can serve as a supporting evidence for change of χ^(3)^, see Figure S5. The incident light source is a solid-state laser at 1030 nm. The spot size at the focus point is 72 μm, which covers a few order/disorder patterns. Therefore, the change of χ^(3)^ could be exhibited in the nonlinear refractive index n_2_, corresponding to third-order susceptibility χ^(3)^ [$n_{2}=\frac{3}{8n_{0}}\chi^{(3)}$].

Based on our measurement, the nonlinear coefficient of the YAG sample is fitted as χ^(3)^=2.96×10^-19^ m^2^/V^2^, which is basically consistent with previous reports [χ^(3)^ = 3.07×10^-19^ m^2^/V^2^, *ref:* *Opt. Express* 2, 11018-11028 (2019)]. According to formula $n_{2}=\frac{\Delta T_{P-V}}{0.406kL_{eff}\left| E \right|^{2}}$, the nonlinear refractive index n_2_ can be derived by determining the transmittance difference ΔT_P-V_ between the peak and valley points of the Z-scan curve. In the formula, *k=2π/λ* and *L_eff_* represents the effective thickness of the sample. Under the same test conditions, *k*, *L_eff_* and |*E|^2^* are equal for both YAG and meta-YAG sample. As a result, the nonlinear coefficient of the meta-YAG sample (containing ordered/disordered regions) is reduced to χ^(3)^≈1.56×10^-19^ m^2^/V^2^.


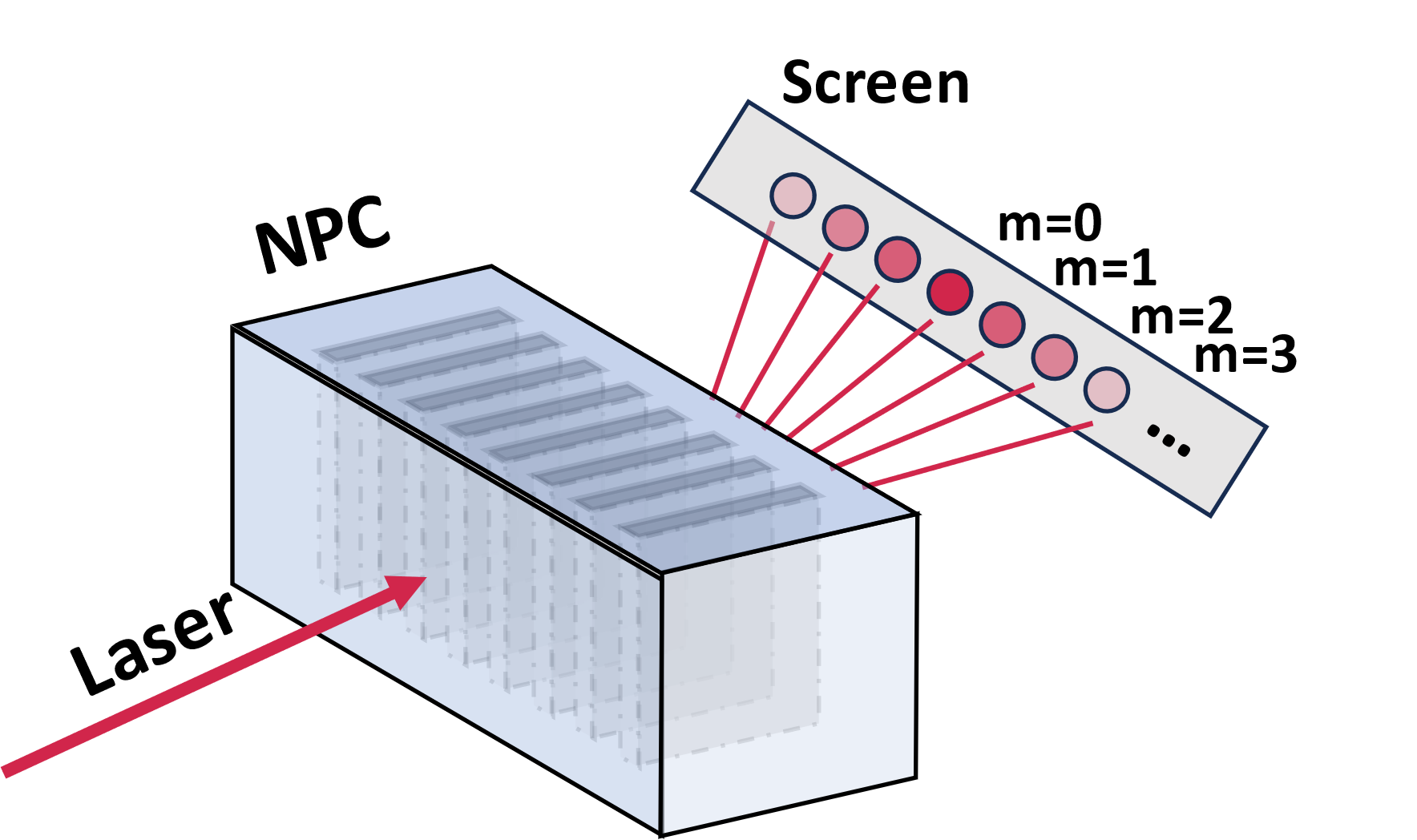


**Supplementary Figure S6.** Schematic diagram of measuring principle of refractive index change. Diffraction occurs when the light is transmitted in the phase grating, and diffraction patterns are formed when the laser of different wavelengths passes through the grating structure in the nonlinear photonic crystal and falls on the optical plate.


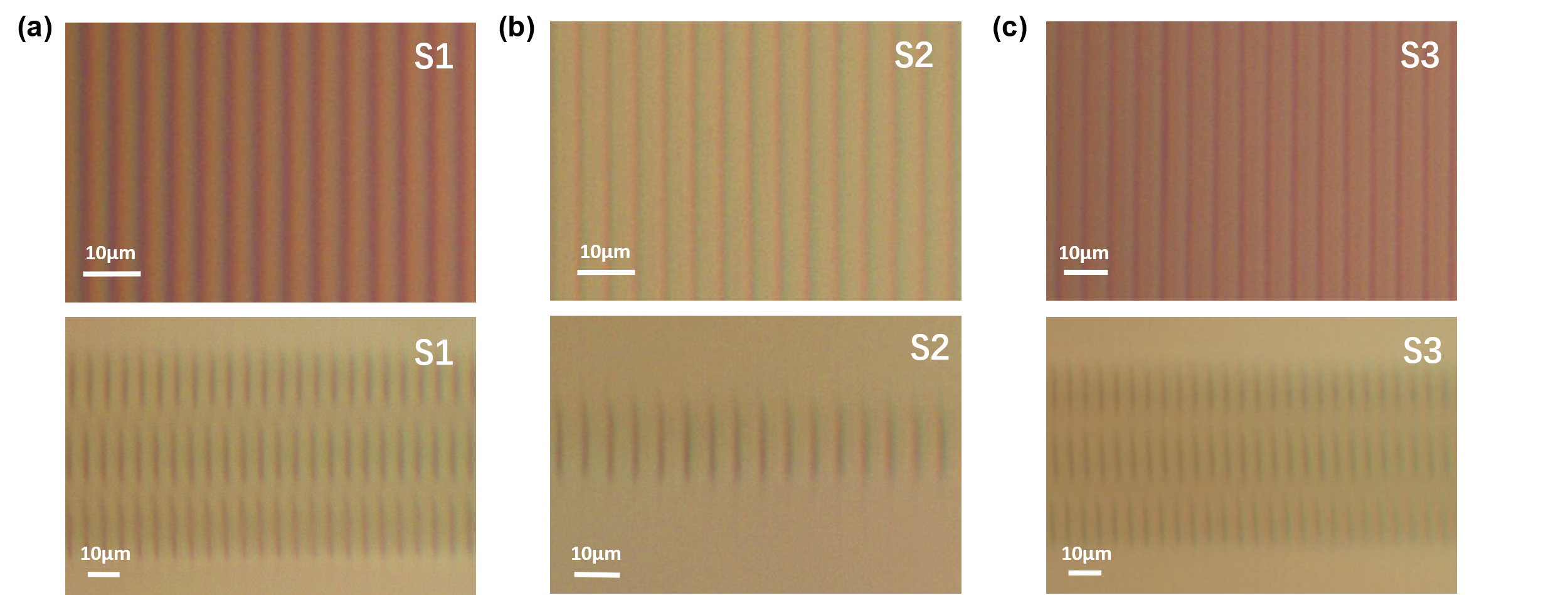


**Supplementary Figure S7.** Micrographs of vertical femtosecond laser processing direction and depth direction of each sample. a) S1 (N=650, P=3). b) S2 (N=1000, P=1). c) S3 (N=1000, P=3).


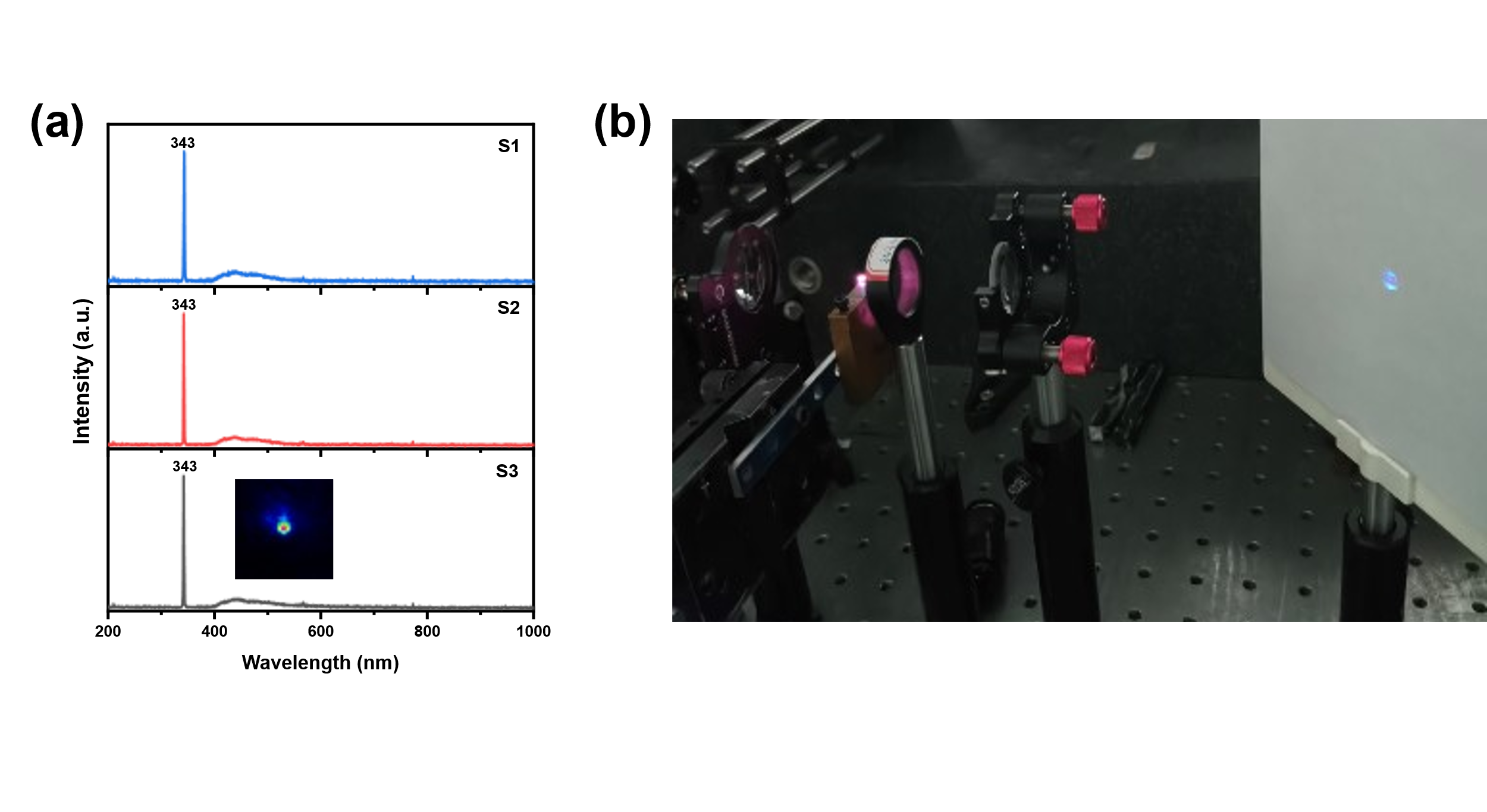


**Supplementary Figure S8.** a) Spectral signals generated by TH of S1-S3 samples and 343 nm TH spot captured by CCD. The broadband spectrum at 400-600 nm is supercontinuum generation under the femtosecond laser illumination. b) Photograph taken under ambient room light showing 343 nm blue light emission from the meta-YAG sample for an incident wavelength at 1030 nm. No visible green light from SHG was observed in the entire system during the experiment.

**
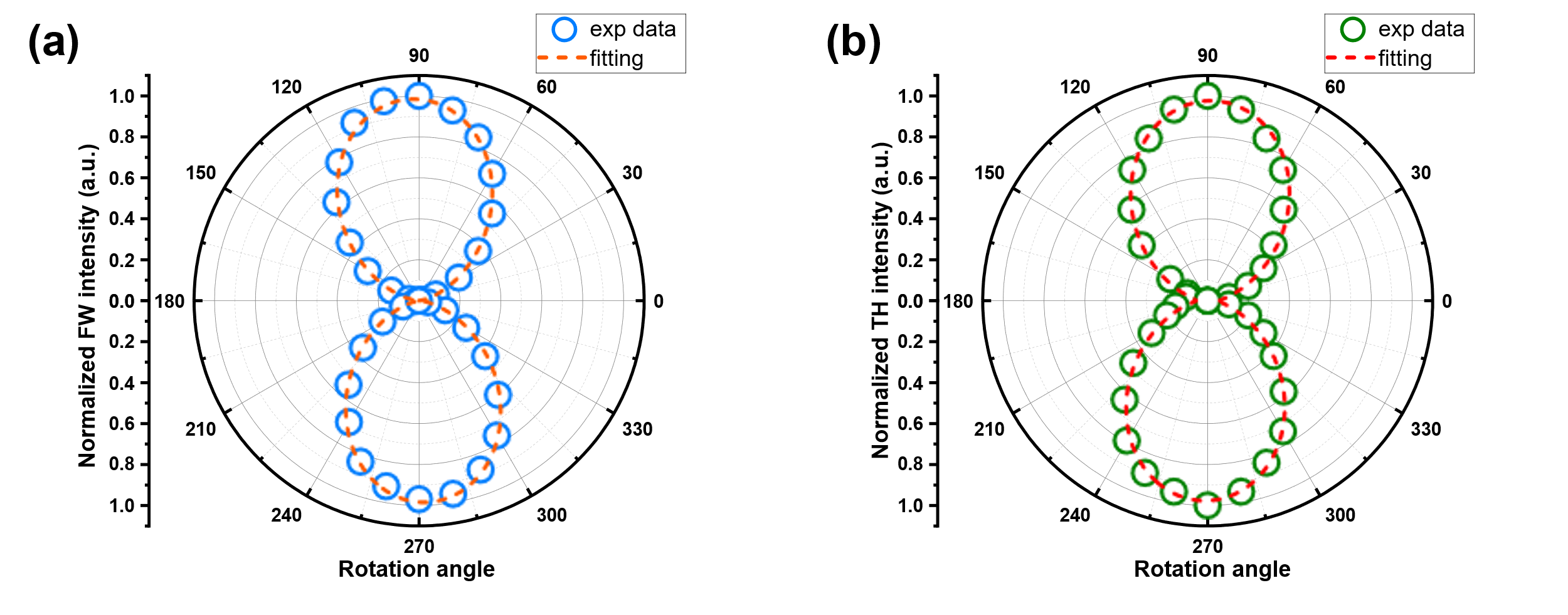
**

**Supplementary Figure S9.** Polarization curve of a) FW at 1030 nm and b) THG at 343 nm in sample S3. The circles represent the experimental test data and the red dashed lines are the fitting results.


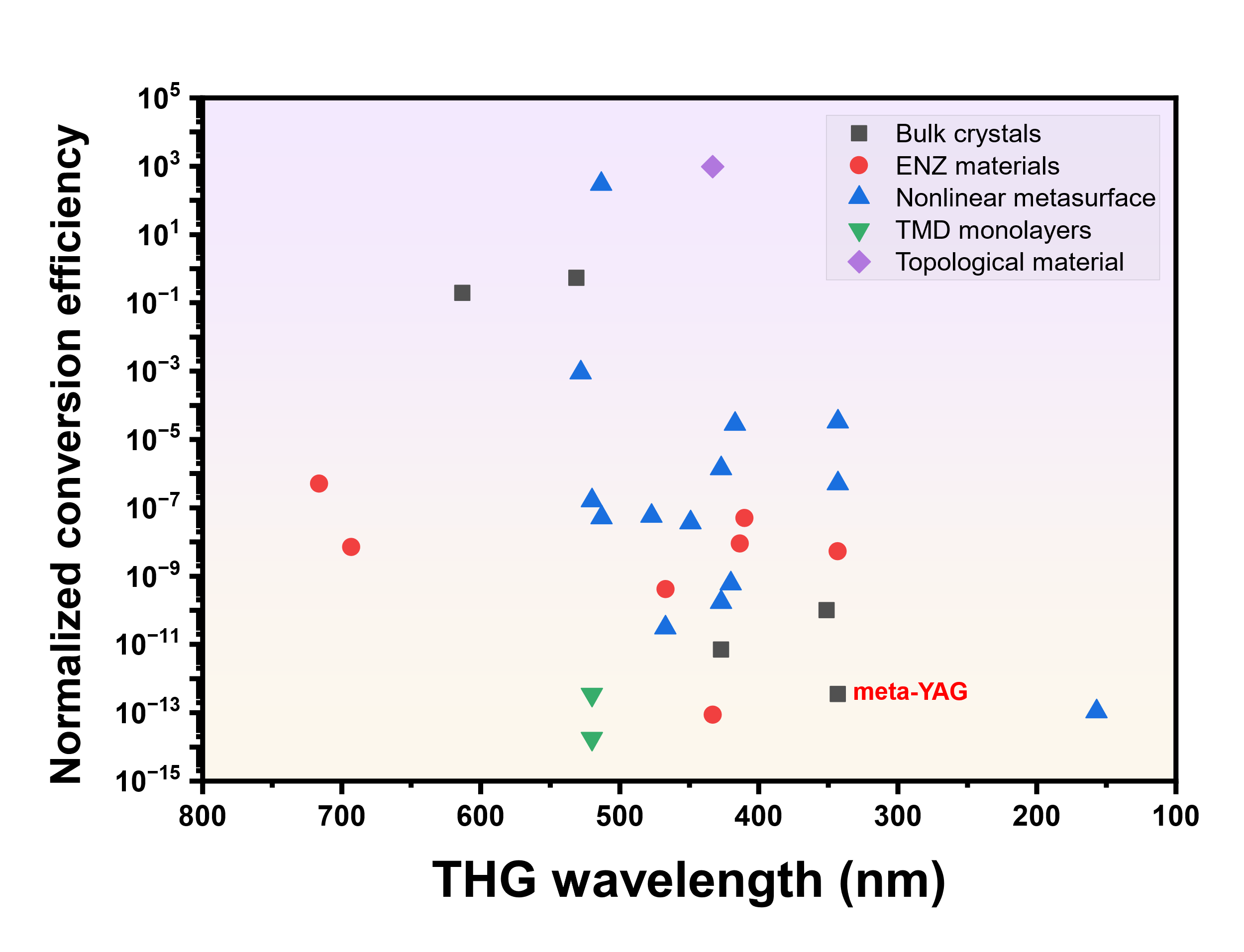


**Supplementary Figure S10.** Comparison of normalized efficiencies of different THG materials.


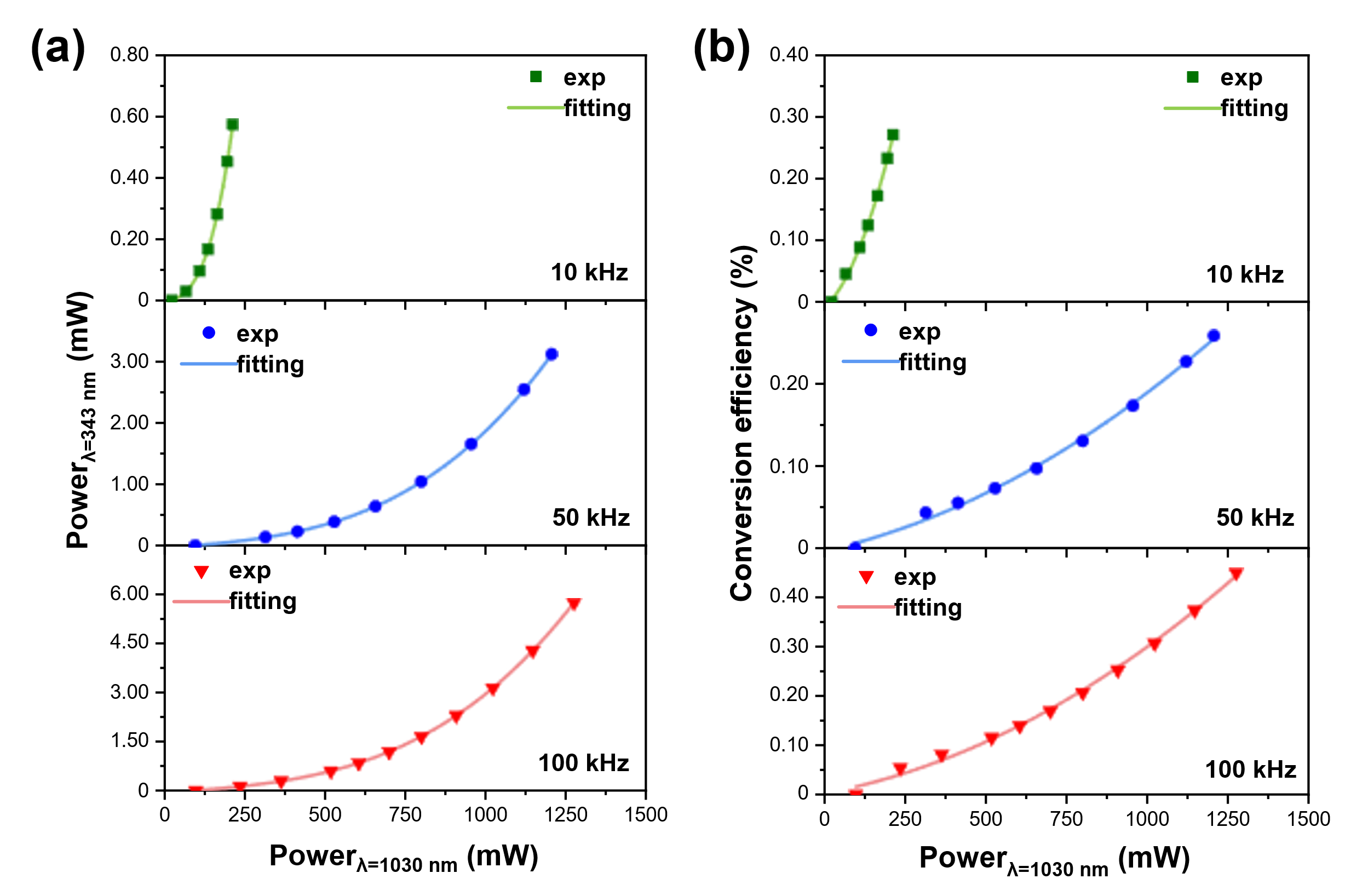


**Supplementary Figure S11.** THG results of meta-YAG at different repetitions. f = 10 kHz, 50 kHz, and 100 kHz.


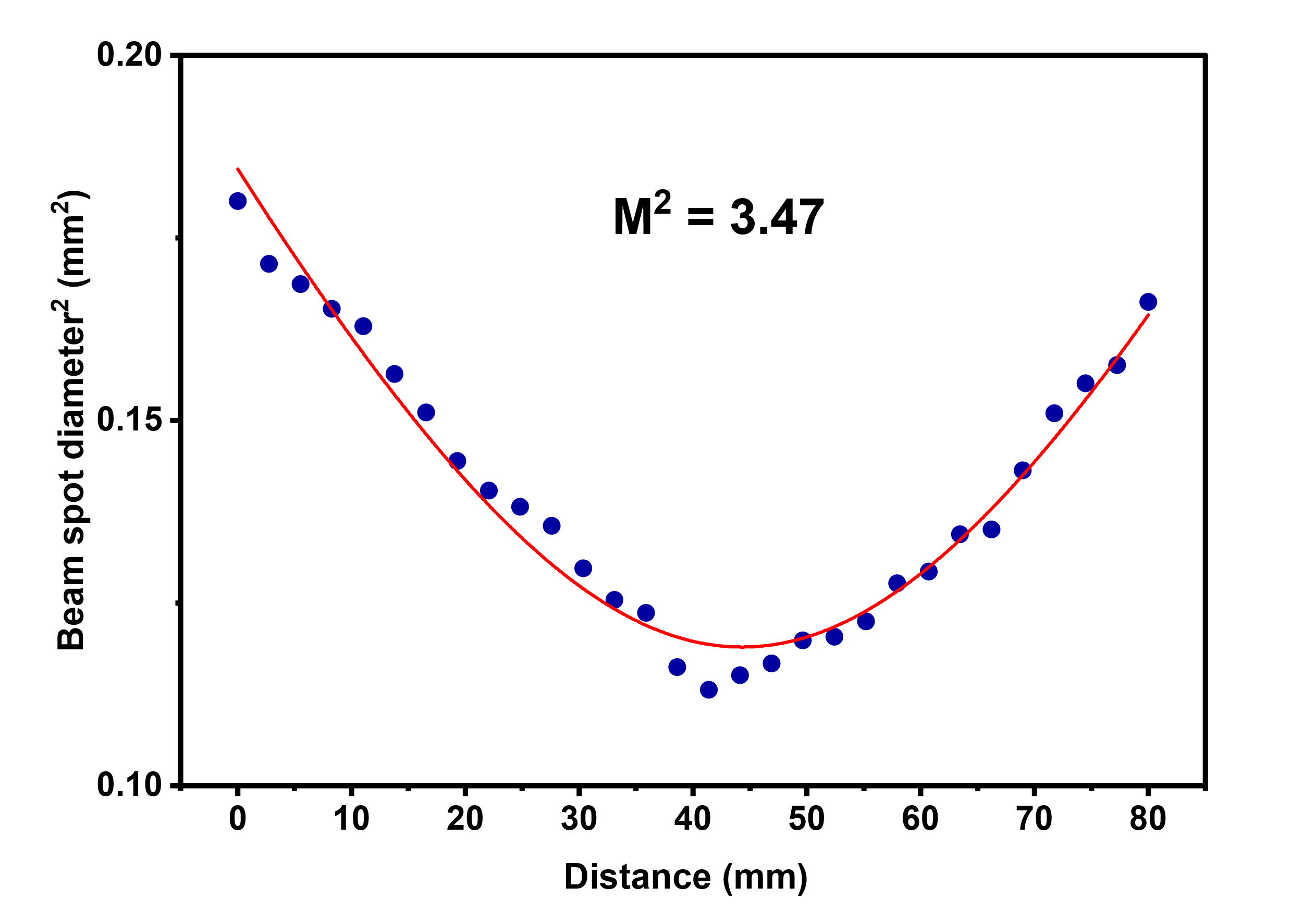


**Supplementary Figure S12.** Beam quality of THG light in meta-YAG.


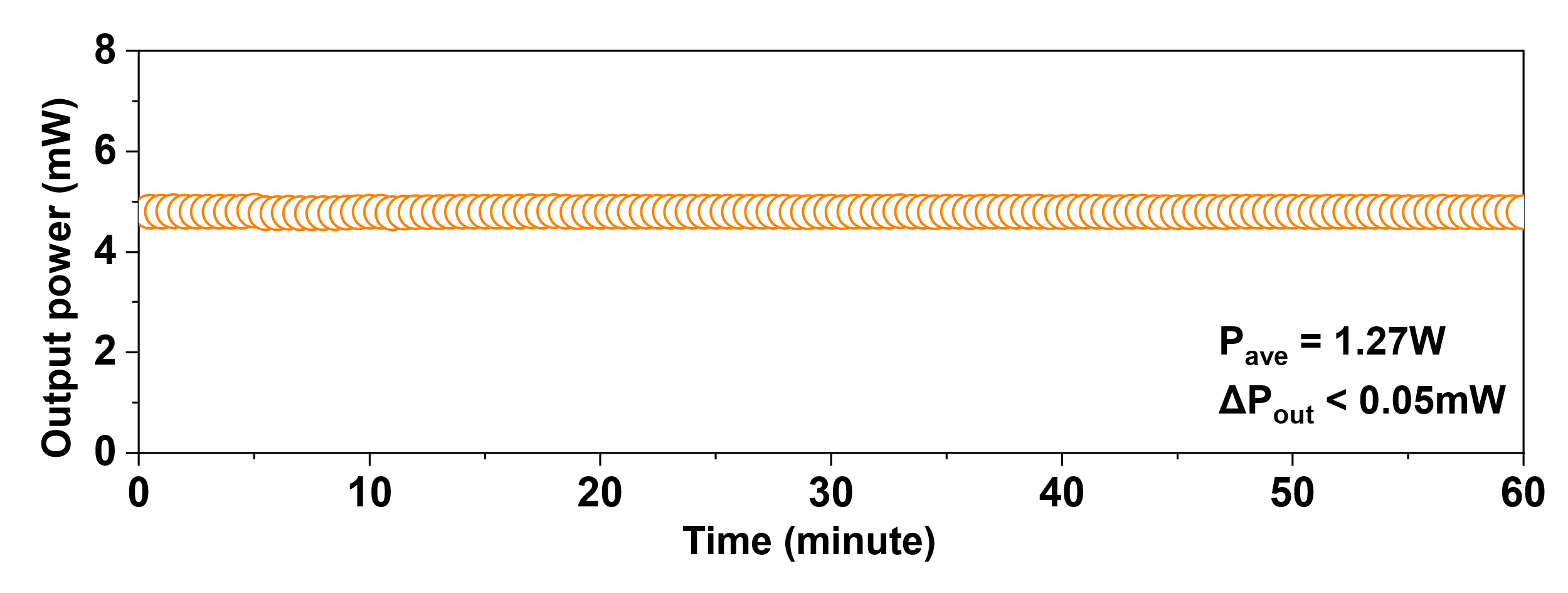


**Supplementary Figure S13.** THG power stability test. The average output power was 4.80 mW, with the maximum power variation less than 0.05 mW within 1 hour.


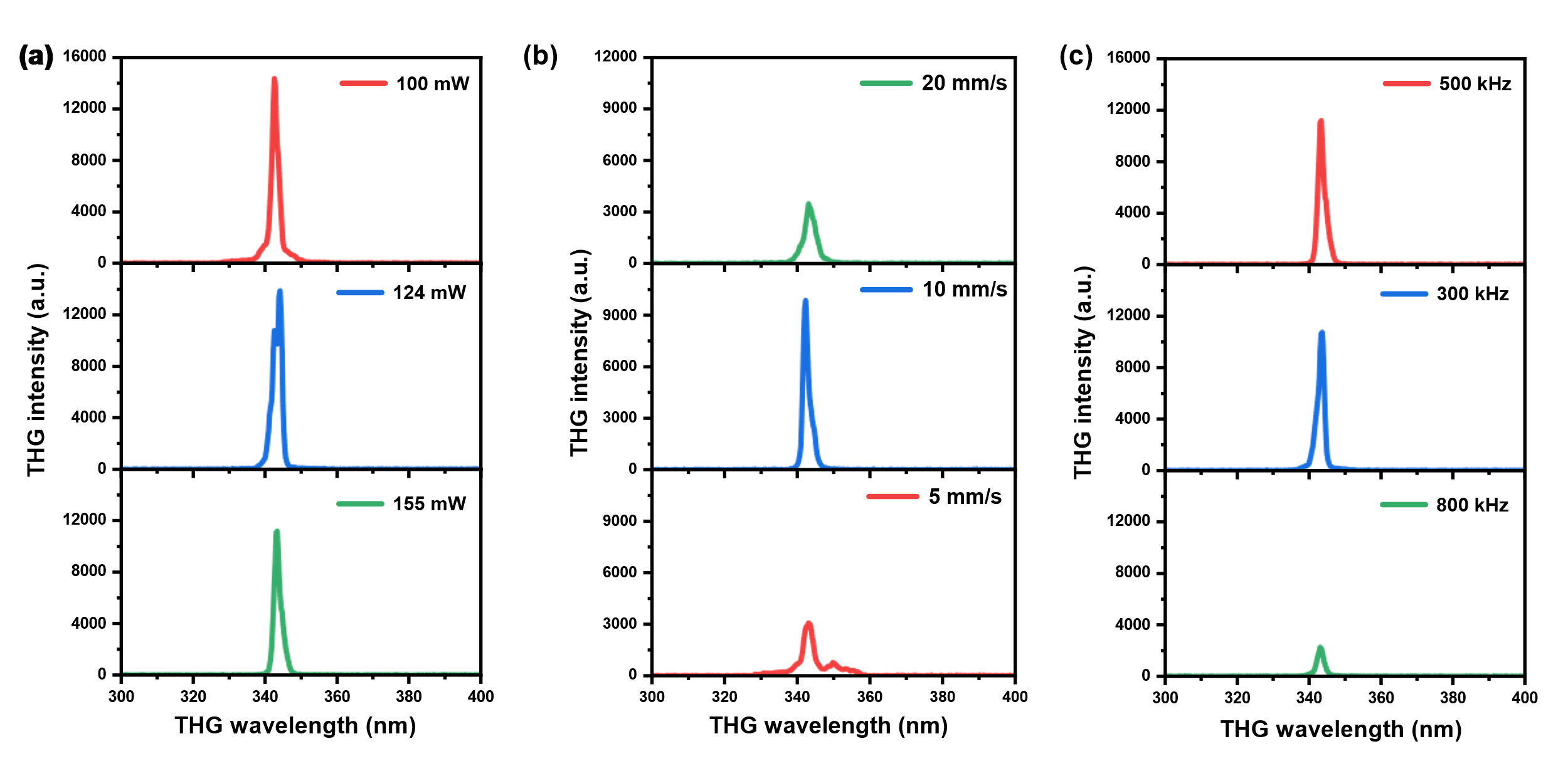


**Supplementary Figure S14.** Comparison of THG intensities under different processing parameters. (a) Processing powers: 100 mW, 124 mW, 155 mW; (b) Scanning speeds: 20 mm/s, 10 mm/s, 5 mm/s; (c) Repetition rates: 300 kHz, 500 kHz, 800 kHz.

**Supplementary Table S1.** A comparison of direct THG in nonlinear optical materials.

| Materials | direction and dimension | χ^(3)^  (m^2^/V^2^) | FW laser parameters | Pump intensity  (GW/cm^2^) | THG wavelength (nm) | Conversion efficiency | Reference |
| --- | --- | --- | --- | --- | --- | --- | --- |
| KTP | Y-cut,  d=8.6 mm | 8.05×10^-22^ | 10 Hz, 15 ps | 0.4 | 531 | 3.4×10^-2^ | [7] |
| α-BBO | type-II PM direction, d=0.5mm | 8.79×10^-23^ | 1 kHz, 120 fs | -- | 266 | 2.5×10^-2^ | [8] |
| TiO_2_ | type-II PM direction,  d=5mm | 9.7×10^−20^ | 10Hz, 15ps | 0.0009 | 613.2 | 1×10^-2^ | [9] |
| Diamond | (100), d=0.5mm | 2.41×10^-19^ | 100 kHz, 60 fs | 1000 | 427 | 7×10^-3^ | [10] |
| **meta-YAG** | (111), d=5mm | 3.07×10^-19^ | 100 kHz, 300 fs | 2350 | 343 | 4.5×10^-3^ | **This work** |
| CaCO_3_ | type-II PM direction, d=20mm | 3×10^-24^ | 5 ps | 100 | 351 | 10^-4^ | [11] |
| TiO_2_ | (100), d=1mm | 5×10^-21^ | 10 Hz, 5 ns | 0.038 | 613.2 | 1.07×10^-5^ | [12] |
| ITO film | thickness  103 nm | 2.48×10^-20^ | 200 kHz, 250 fs | 51.4 | 343 | 7.05×10^-4^ | [13] |
| AZO monolayer | thickness  100 nm | 4.62×10^−22^ | 1 kHz, 100 fs | 6.37×10^-5^ | 516.7 | 2.8×10^-4^ | [14] |
| CdO film | thickness  75 nm | -- | 1 kHz, 60 fs | 11.3 | 693 | 10^-5^ | [15] |
| AZO film | thickness  300 nm | -- | 1 kHz, 85 fs | 475 | 433 | 9.2×10^-6^ | [16] |
| ITO film | thickness  33 nm | -- | 100 kHz, 50 fs | 20 | 466.7 | 3.3×10^-6^ | [17] |
| Si/Dy:CdO multilayer | thickness  100nm | 10^−20^ | 150 fs | 1 | 716 | 5×10^-7^ | [18] |
| ITO film | thickness  350 nm | 1.68×10^−18^ | 1 kHz, 35 fs | -- | 416 | 3.8×10^-7^ | [19] |
| ITO-GaP | -- | 3.84×10^−20^ | 100 kHz, 220 fs | -- | 450 | 1.97×10^-7^ | [20] |
| ITO film | thickness  30 nm | 3×10^−21^ | 1 kHz, 50-102 fs | 1-2 | 413.3 | 3×10^-8^ | [21] |
| ITO  nanolayers | thickness  20 nm | -- | 80 MHz, 150 fs | 0.45 | 410 | 4.5×10^-9^ | [22] |
| silicon nano-disk | thickness  220 nm | 3.49×10^-18^ | 2.7kHz, 200fs | ~5.5 | 420 | ~10^-7^ | [23] |
| Nodal-Line semimetal ZrSiS | -- | 7.47×10^−12^ | 1kHz, 120fs | 7.6×10^-3^ | 433.3 | 4.3×10^-4^ | [24] |
| dielectric metasurface | thickness  580 nm | -- | 200 kHz, 250 fs | 5.31 | 343 | 7.71×10^-5^ | [25] |
| dielectric metasurface | thickness  400 nm | -- | 200 kHz, 250 fs | 0.7 | 343 | 1.13×10^-5^ | [26] |
| silicon metasurface | thickness  350 nm | 2.45×10^-19^ | 80 MHz, 140~200 fs | 23.3 | 427 | 2.2×10^-6^ | [27] |
| all-dielectric metasurface | -- | 2.79×10^-18^ | 80MHz, ~200fs | 0.4 | 417 | 1.8×10^-6^ | [28] |
| silicon metasurface | thickness  120 nm | 2.79×10^-18^ | 80MHz, ~250fs | ~3.2 | 449 | 1.2×10^-6^ | [29] |
| all-dielectric metasurface | thickness  230 nm | -- | 1kHz, 100fs | ~33 | 467 | 1.1×10^-6^ | [30] |
| dielectric metasurface | thickness  538 nm | 2.45×10^-19^ | 80 MHz, ~200 fs | 2.6 | 477 | ~10^-6^ | [31] |
| dielectric metasurface | thickness  500 nm | -- | -- | 0.1 | 528 | 9×10^-7^ | [32] |
| silicon metasurface | thickness  590 nm | 2.45×10^-19^ | 80 MHz, 80 fs | 1.2 | 520 | 2.8×10^-7^ | [33] |
| complementary Silicon | thickness  205 nm | -- | 82 MHz, ~200 fs | ~0.5 | 427 | 1.76×10^-7^ | [34] |
| Resonant membrane metasurfaces | thickness  1 μm | -- | 1.49 kHz, ~800 fs | 0.3 | 1320 | 6.4×10^-9^ | [35] |
| silicon metasurfaces | thickness  300 nm | 2.45×10^-19^ | 10 kHz, 20 ns | 0.00549 | 513.4 | 10^-5^-10^-4^ | [36] |
| dielectric nanofilm | thickness  300 nm | 2.8×10^-22^ | 1 kHz; 100 fs | 209 | 146-157 | 1×10^-6^ | [37] |
| MoS_2_ monolayer | thickness  0.65 nm | 2.4×10^-19^ | 50 MHz, 150 fs | -- | 520 | 6.6×10^-10^ | [38] |
| MoS_2_ monolayer | thickness  0.65 nm | 2.4×10^−19^ | 50 MHz, 150 fs | 30 | 520 | 4.76×10^-10^ | [39] |
| MoS_2_ monolayer | thickness  ~0.7 nm | 1.7×10^−28^ | 89 MHz, 150 fs | 10 | 520 | 3.4×10^-10^ | [40] |
| WS_2_ monolayer | thickness  ~0.7 nm | 3.6×10^-19^ | 50 MHz, 150 fs | -- | 520 | 2.8×10^-10^ | [38] |
| ReS_2_ monolayer | thickness  0.73 nm | ~10^−18^ | 80 MHz, 250 fs | -- | 505 | 2.3×10^-10^ | [41] |
| hBN | thickness  37 nm | (8.4±0.5) ×10^-21^ | 80 MHz, 150 fs | 33 | 360 | -- | [42] |
| hBN | thickness  10-50 mm | -- | 1 kHz, 70-120 fs | 0.25 | 2430 | -- | [43] |

**Supplementary Table S2.** Specific parameters of femtosecond laser processing

| Sample | Period (μm) | Number of periods | Number of layers | Distance between machining position and upper surface (mm) | Wavelength  (nm) | repetition rate  (kHz) | pulse width  (fs) | Power (mW) | Accuracy (μm) | Monolayer depth (μm) | Interlamellar spacing (μm) |
| --- | --- | --- | --- | --- | --- | --- | --- | --- | --- | --- | --- |
| S1 | 5.28 | 650 | 3 | 0.75 | 1030 | 500 | 200 | 100 | 1.75 | 20 | 5 |
| S2 |  | 1000 | 1 |  |  |  |  |  |  |  | -- |
| S3 |  | 1000 | 3 |  |  |  |  |  |  |  | 5 |

**References**

[1] J. A. Armstrong, N. Bloembergen, J. Ducuing, et al, *Phys. Rev.* **1962**, *127*, 1918.

[2] N. Bloembergen, P. S. Pershan, *Phys. Rev.* **1962**, *128*, 606.

[3] N. Bloembergen, Y. R. Shen, *Phys. Rev.* **1964**, *133*, A37.

[4] Y. R. Shen, Principles of Nonlinear Optics, *Wiley.* **1984**.

[5] R. W. Boyd, A. L. Gaeta, E. Giese, Handbook of Atomic, Molecular, and Optical Physics, *Springer.* **2008**.

[6] J. Hrabovský, M. Kučera, L. Paloušová, et al, *Opt. Mater. Express.* **2021**, *11*, 1218.

[7] A. Vernay, V. Boutou, C. Félix, et al, *Opt. Express.* **2021**, *29*, 22266.

[8] M. Shi, G. Zhang, B. Li, et al, *Opt. Lett.* **2018**, *43*, 1734.

[9] A. Borne, P. Segonds, B. Boulanger, et al, *Opt. Mater. Express*. **2012**, *2*, 1797.

[10] A. Abulikemu, M. J. Hase, *Opt. Mater. Express.* **2023**, *13*, 916.

[11] A. Penzkofer, F. Ossig, P. Qiu, *Appl. Phys. B,* **1988**, *47*, 71.

[12] F. Gravier, B. Boulanger, *Opt. Express.* **2006**, *14*, 11715.

[13] W. Tian, F. Liang, D. Lu, et al, *Photonics Res*. **2021**, *9*, 317.

[14] J. Wu, Z. Xie, H. Fu, et al, *Results Phys*. **2021**, *24*, 104086.

[15] Y. Yang, J. Lu, A. Manjavacas, et al, *Nat. Physics*. **2019**, *15*, 1022.

[16] W. Jaffray, F. Belli, S. Stengel, et al, *Adv. Opt. Mater.* **2024**, 2401249.

[17] T. LuK, D. De Ceglia, S. Liu, et al, *Appl. Phys. Lett.* **2015**, *106*, 151103.

[18] M. Vincenti, D. De Ceglia, M. Scalora. *Opt. Express*. **2020**, *28*, 31180.

[19] J. Li, J. Huang, *Opt. Commun.* **2024**, *561*, 130545.

[20] R. Tirole, B. Tilmann, L. Menezes, et al, *Adv. Opt. Mater*. **2024**, *12*, 2302069.

[21] L. Rodríguez-Suné, M. Scalora, A. Johnson, et al, *APL Photonics*. **2020**, *5*, 010801.

[22] T Shubitidze, S Chawla, L Dal Negro. *APL Photonics.* **2025**, 10, 026110.

[23] M. Shcherbakov, D. Neshev D N, B. Hopkins, et al, *Nano Lett*. **2014**, *14*, 6488.

[24] S. Chi, F. Liang, H. Chen, et al, *Adv. Mater.* **2020**, *32*, 1904498.

[25] Z. Wang, Q. Zhao, W. Tang, et al, *Appl. Phys. Lett.* **2024**, *124*, 101101.

[26] W. Tang, Q. Zhao, Z. Wang, et al, *Opt. Lett.* **2024**, *49*, 1169.

[27] A. Liu, J. Hsieh, K. Lin, et al, *Adv. Opt. Mater.* **2023**, *11*, 2300526.

[28] G. Yang, S. Dev, M. Allen, et al, *Nano Lett.* **2022**, *22*, 2001.

[29] Y. Yang, W. Wang, A. Boulesbaa, et al, *Nano Lett.* **2015**, *15,* 7388.

[30] M. Shcherbakov, D. Neshev, B. Hopkins, et al, *Nano Lett.***2014**, *14*, 6488.

[31] K. Koshelev, Y. Tang, K. Li, et al, *ACS Photonics* **2019**, *6*, 1639.

[32] Z. Liu, Y. Xu, Y. Lin, et al, *Phys. Rev. Lett.* **2019**, *123*, 253901.

[33] D. Hähnel, C. Golla, M. Albert, et al, *Light: Sci. Appl.* **2023**, *12*, 97.

[34] S. Chen, M. Rahmani, K. Li, et al, *ACS Photonics*, **2018**, *5*, 1671.

[35] P Tonkaev, F Richter, I Toftul, et al. *Nat. Commun.* **2025**, *16*, 11571.

[36] K Sun, K Wang, W Wang, et al. *Newton*. **2025**, *1*, 100057.

[37] K Konishi, D Akai, Y Mita, et al. *APL Photonics*. **2020**, *5*, 066103.

[38] A Autere, H Jussila, A Marini, et al. *Phys. Rev. B*. **2018**, *98*, 115426.

[39] A. Säynätjoki, L. Karvonen, H. Rostami, et al, *Nat. Commun.* **2017**, *8*, 893.

[40] R. Woodward, R. Murray, C. Phelan, et al. *2D Mater.* **2017**, *4*, 011006.

[41] Q. Cui, R. Muniz, J. Sipe, et al. *Phys. Rev. B*, **2017**, *95*, 165406.

[42] A Popkova, I Antropov, J Fröch, et al. *ACS Photonics*, **2021**, *8*, 824.

[43] J Ginsberg, M Jadidi, J Zhang, et al. *Nature Communications*, **2023**, *14*, 7685.
